# Supplementary material for: Characterisation of TbSmee1 suggests endocytosis allows surface-bound cargo to enter the trypanosome flagellar pocket
Source: J Cell Sci. 2023 Oct 26;136(20):jcs261548. doi: 10.1242/jcs.261548 (PMC10652038; doi:10.1242/jcs.261548)
Supplement: Supplementary information [file joces-136-261548-s1.pdf]

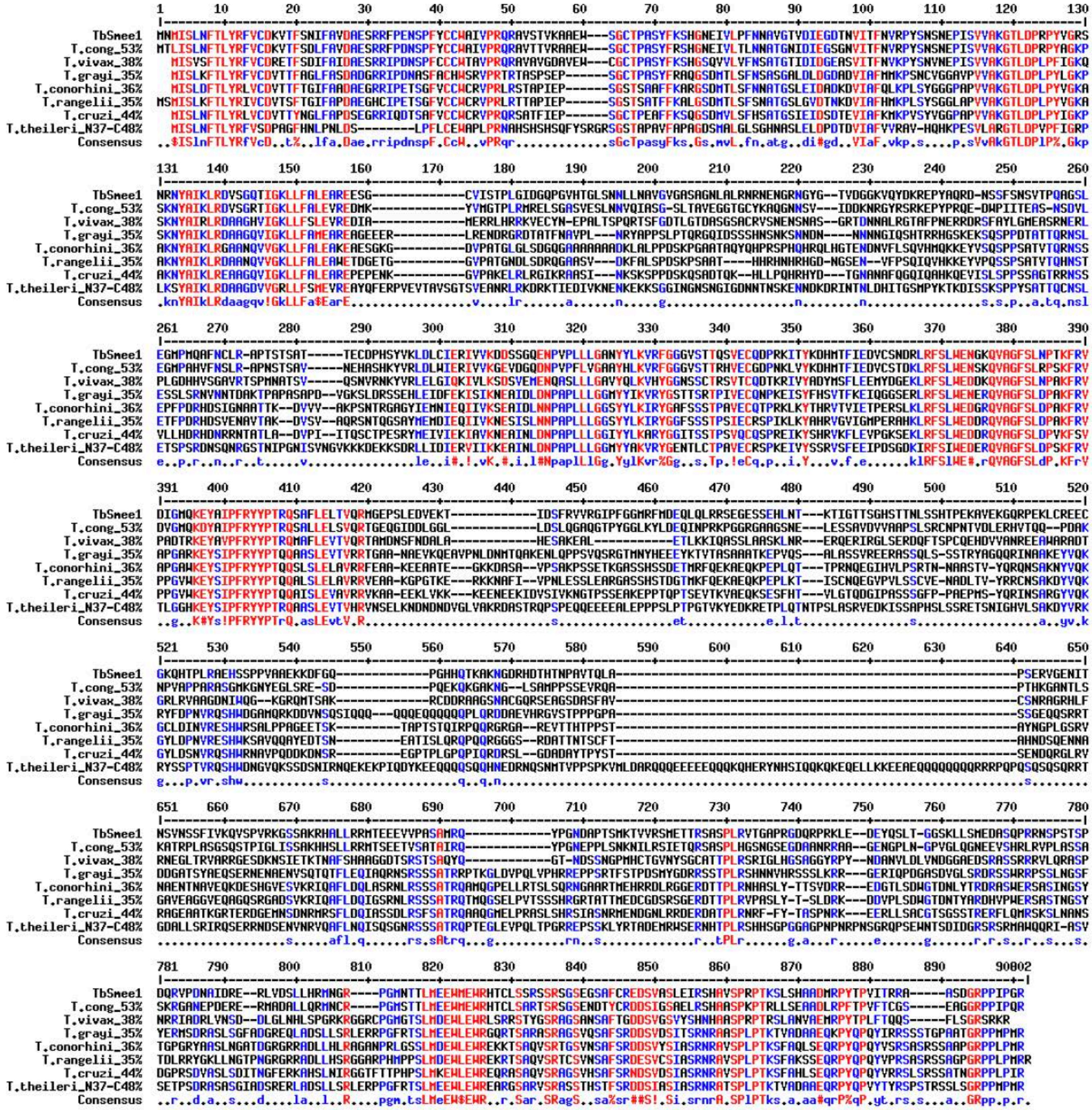

Fig. S1. The TbSmee1 primary structure contains three blocks of highly-conserved sequence. Multiple sequence alignment of Trypanosoma brucei Smee1 (TbSmee1) and homologous proteins from other trypanosome species, generated using MultAlin (Corpet, 1988) with default parameters. Amino acid numbers are indicated in black numerals above the alignment. Moderately (50-90%) conserved residues are highlighted in blue. Highly-conserved (>90%) or completely conserved residues are highlighted in red. The consensus sequence is shown below the alignment. Abbreviations: TbSmee1, Trypanosoma brucei Smee1; T.cong, Trypanosoma congolense; T.vivax, Trypanosoma vivax; T.grayi, Trypanosoma grayi. T.conorhini, Trypanosoma conorhini; T.rangelii, Trypanosoma rangelii; T.cruzi, Trypanosoma cruzi; T.theileri, Trypanosoma theileri. The % sequence identity of each homologue to TbSmee1 is indicated after the name.

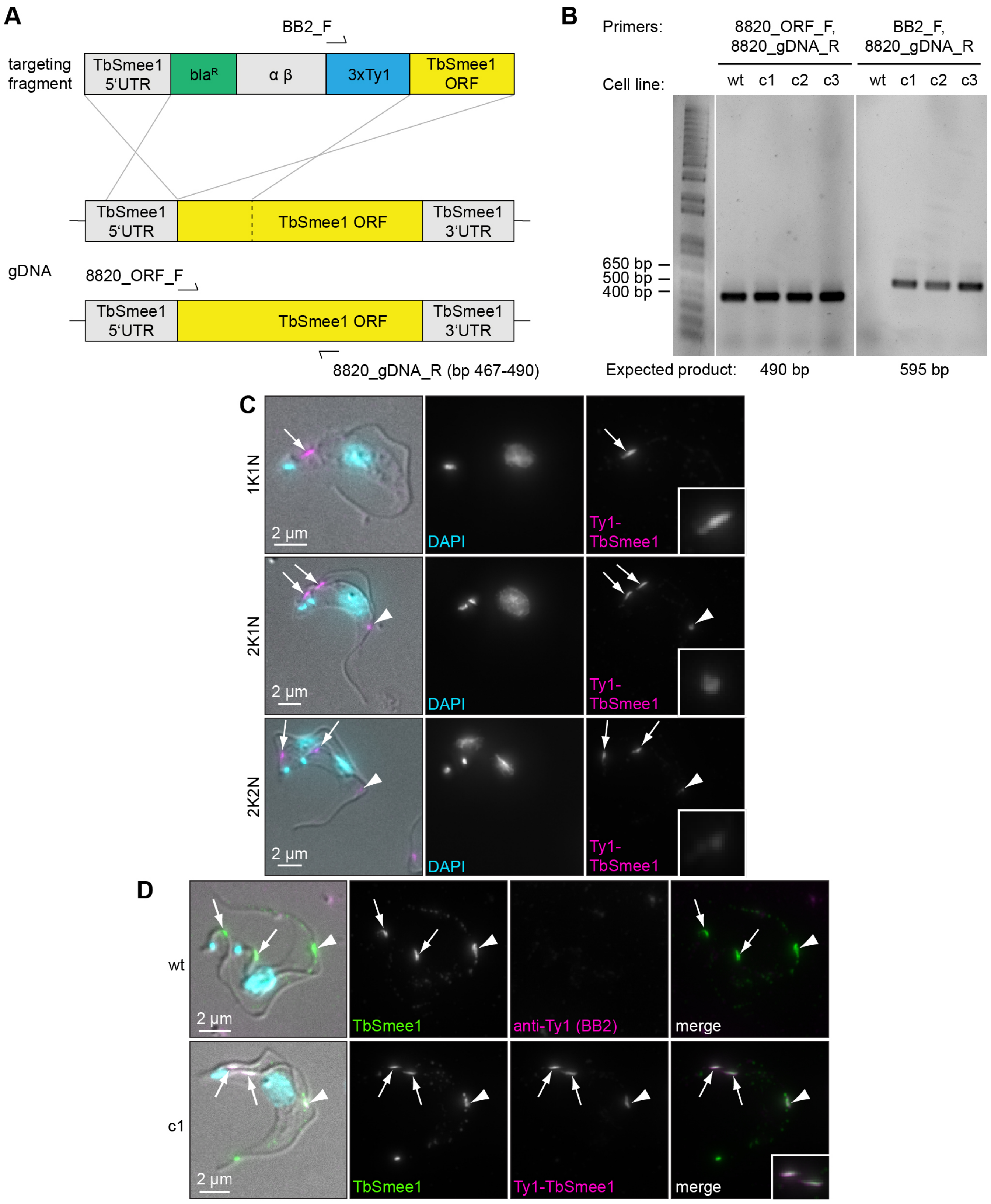

Journal of Cell Science • Supplementary information

Fig. S2. Generation of Ty1-Tb5mee1 endogenous replacement cell line. (A) Schematic showing the in situ tagging procedure and annealing sites for PCR primers. The two endogenous alleles of TbSmee1 in the gDNA are shown in yellow, flanked by 5'UTR and 3'UTR sequences. T. brucei cells were transfected with a targeting fragment with homology arms for recombination with the 3' end of the 5'UTR and 5' end of the ORF. The targeting fragment contained a blasticidin resistance gene (blaR), the intergenic region from the alpha/beta tubulin locus ($\alpha\beta$) and a 3xTy1 epitope tag preceded by an ATG start codon. Homologous recombination removed the endogenous ATG start codon of the TbSmee1 ORF. (B) Confirmation of targeting fragment integration at the endogenous TbSmee1 locus by PCR analysis of genomic DNA. Genomic DNA from wild-type (wt) and candidate Ty1-TbSmee1 clones (c1, c2, c3) was analysed by PCR. Left panel: positive control using 8820_0RF_F and 8820_gDNA_R primers; 490 bp product expected. Right panel: integration test using BB2_F and 8820_gDNA_R primers. A product is only expected if the 3xTy1 sequence has integrated upstream of the TbSmee1 ORF (see primer annealing sites in panel A). Two independent experiments were carried out, each using all three separate clones. (C) Ty1-TbSmee1 displays the same localisations through the cell cycle as endogenous TbSmee1. Detergent-extracted cells were fixed with methanol and labelled with anti-Ty1 antibodies; DNA was stained using DAPI. Exemplary cells from the three main cell cycle states (1K1N, 2K 1N, 2K2N) are shown. Maximum-intensity z-projections of the fluorescence channels are shown, together with a single DIC z-slice overlay. The Ty1-TbSmee1 signal is shown in magenta in the overlay and highlighted with arrows. Arrowheads indicate the Ty1-TbSmee1 present at the tip of the new FAZ. Insets show an enlarged view of the TbSmee1 signal from the hook complex or FAZ tip. Multiple (n>3) independent experiments were carried out using three separate clones. (D) The anti-Ty1 signal is specific for Ty1-TbSmee1. Wild-type (wt) and Ty1-TbSmee1 cells were extracted with detergent, fixed with methanol, and labelled with anti-TbSmee1 and anti-Ty1 antibodies. Hook complex (arrows) and FAZ tip (arrowheads) localisations are indicated. No anti-Ty1 signal was seen in wild-type cells; strong overlap between the anti-Ty1 and anti-TbSmee1 signals was seen in the Ty1-TbSmee1 cells. Maximum-intensity z-projections of the fluorescence channels are shown , together with a single DIC z-slice overlay.

Journal of Cell Science • Supplementary information

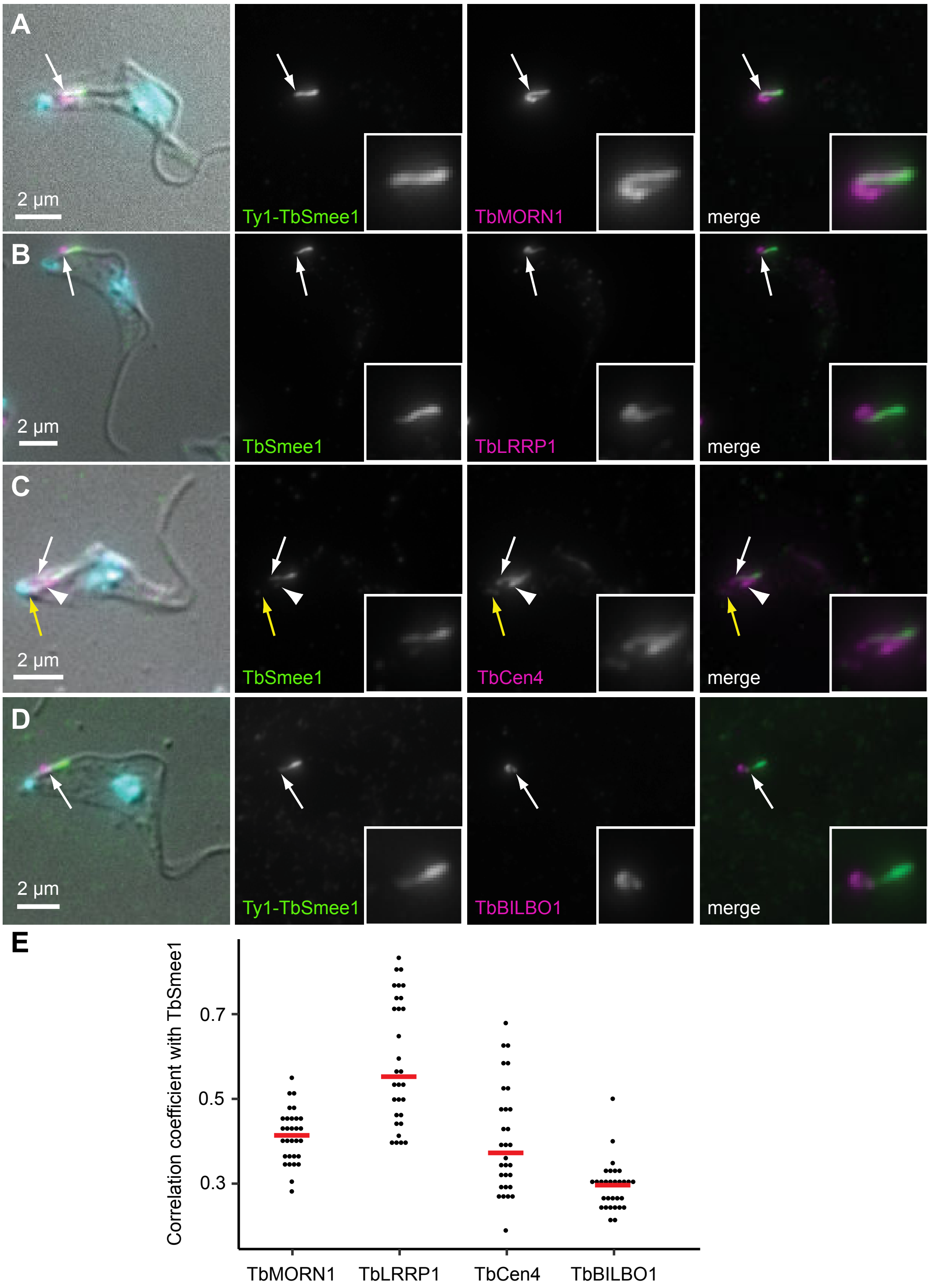

Fig. S3. TbSmee1 is localised to the shank part of the hook complex. Bloodstream form trypanosomes were extracted with non-ionic detergent, fixed, and labelled with the indicated antibodies. Either wild-type or Ty1-TbSmee1 cells were used. Insets show an enlarged view of the hook complex region. (A) TbSmee1 overlaps with the shank part of the hook complex protein TbMORN1 (arrow). (B) TbSmee1 overlaps with the shank part of the hook complex protein TbLRRP1 (arrow). (C) TbSmee1 partially overlaps with TbCen4. TbCen4 is present at the basal and probasal bodies (yellow arrow), centrin arm (arrowhead), and a small additional projection (white arrow). (D) TbSmee1 does not overlap with the flagellar pocket collar protein TbBILBO1 (arrow). (E) Summary of measured correlation coefficients for each of the colabelling experiments; red bars show median values. TbSmee11 showed a moderate correlation with TbMORN1 (0.41) and TbLRRP1 (0.55) and a weak correlation with TbCen4 (0.37) TbBILBO1 (0.3). Each dot represents a single cell in the 1K1N stage (N = 30). All fluores-cence images are maximum intensity z-projections, and an overlay with a single DIC section is shown. Overlap was manually confirmed in single z-slices. Results were obtained from multiple (n>3) independent experiments; exemplary images are shown.

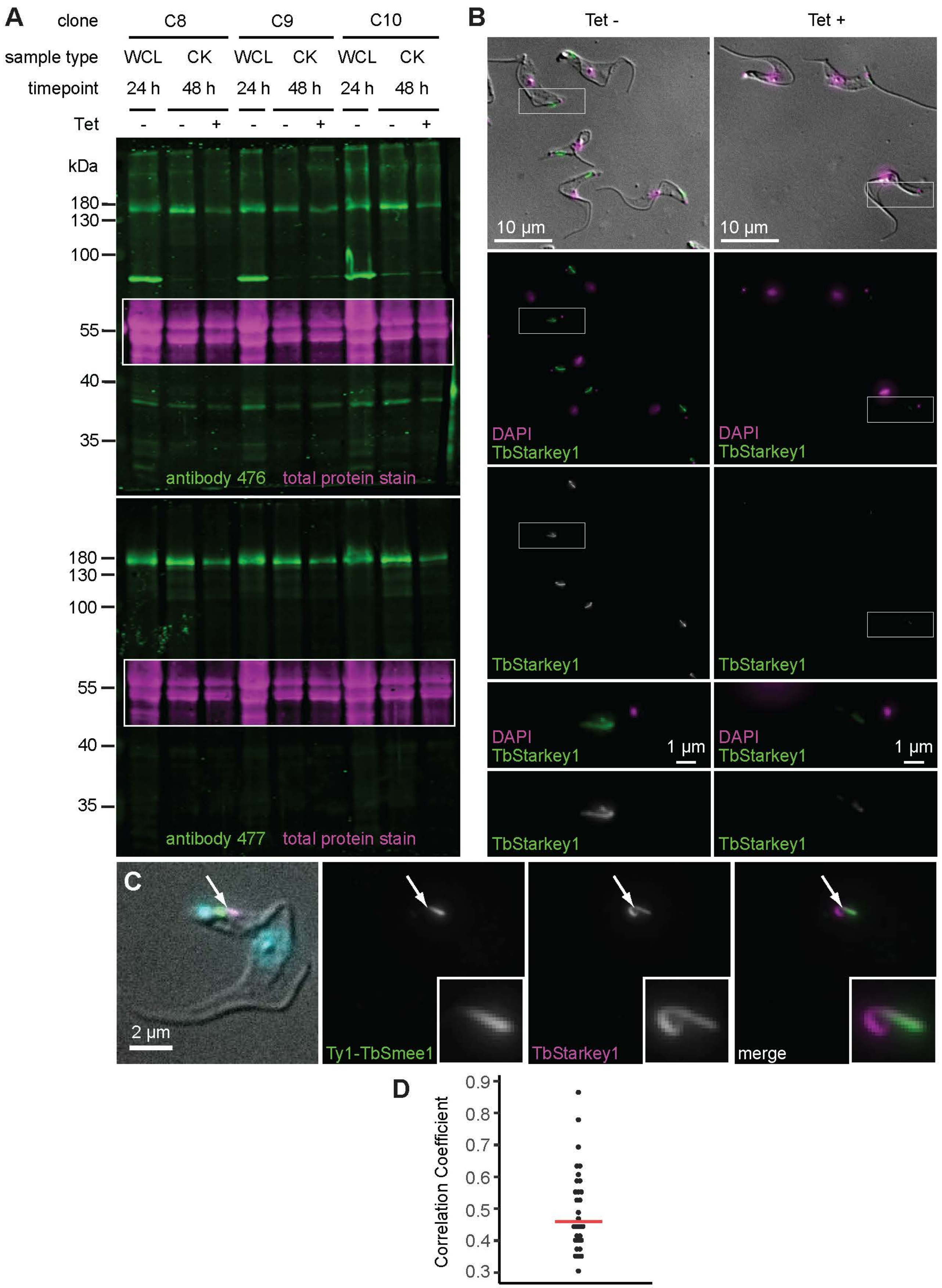

Journal of Cell Science • Supplementary information

Fig. S4. Validation of anti-TbStarkey1 antibodies. (A) Validation of anti-TbStarkey1 antibody specificity by immunoblotting. Three separate TbStarkey1 RNAi clones (C8, C9, C10) were analysed by immunoblotting. Both whole-cell lysates (WCL) and detergent-extracted cytoskeleton (CK) samples were obtained at the indicated timepoints (24 h, 48 h) from control (Tet-) and induced (Tet+) cells. The samples were immunoblotted using two separate anti-TbStarkey1 affinity-purified antibodies (antibody 476, 477). Both antibodies recognised a >130 kDa protein whose abundance was depleted after 48 h of RNAi. The 476 antibody additionally showed a significant cross-reaction with a protein of <100 kDa in WCL but not CK samples. A section of the total protein stain of each membrane is shown as an inset (magenta). (B) Validation of anti-TbStarkey1 antibody specificity by immunofluorescence microscopy. Control (Tet-) and TbStarkey1-depleted (Tet+) RNAi cells were extracted with non-ionic detergent, fixed with methanol, and labelled with anti-TbStarkey1 antibodies (green); DNA was stained using DAPI (magenta). TbStarkey1 localised to the hook complex, and signal was lost upon depletion. The boxed areas are shown enlarged in the bottom panels. Identical results were obtained using both anti-TbStarkey1 antibodies; exemplary images using the 477 antibodies are shown. (C) TbSmee1 overlaps with the shank part of the hook complex protein TbStarkey1 (arrow). All fluorescence images are maximum intensity projections, and an overlay with a single DIC section is shown. Overlap was manually confirmed in single z-slices. Results were obtained from multiple (n>3) independent experiments; exemplary images are shown. (D) Summary of measured correlation coefficients. Each dot represents a single cell; red lines show median values.

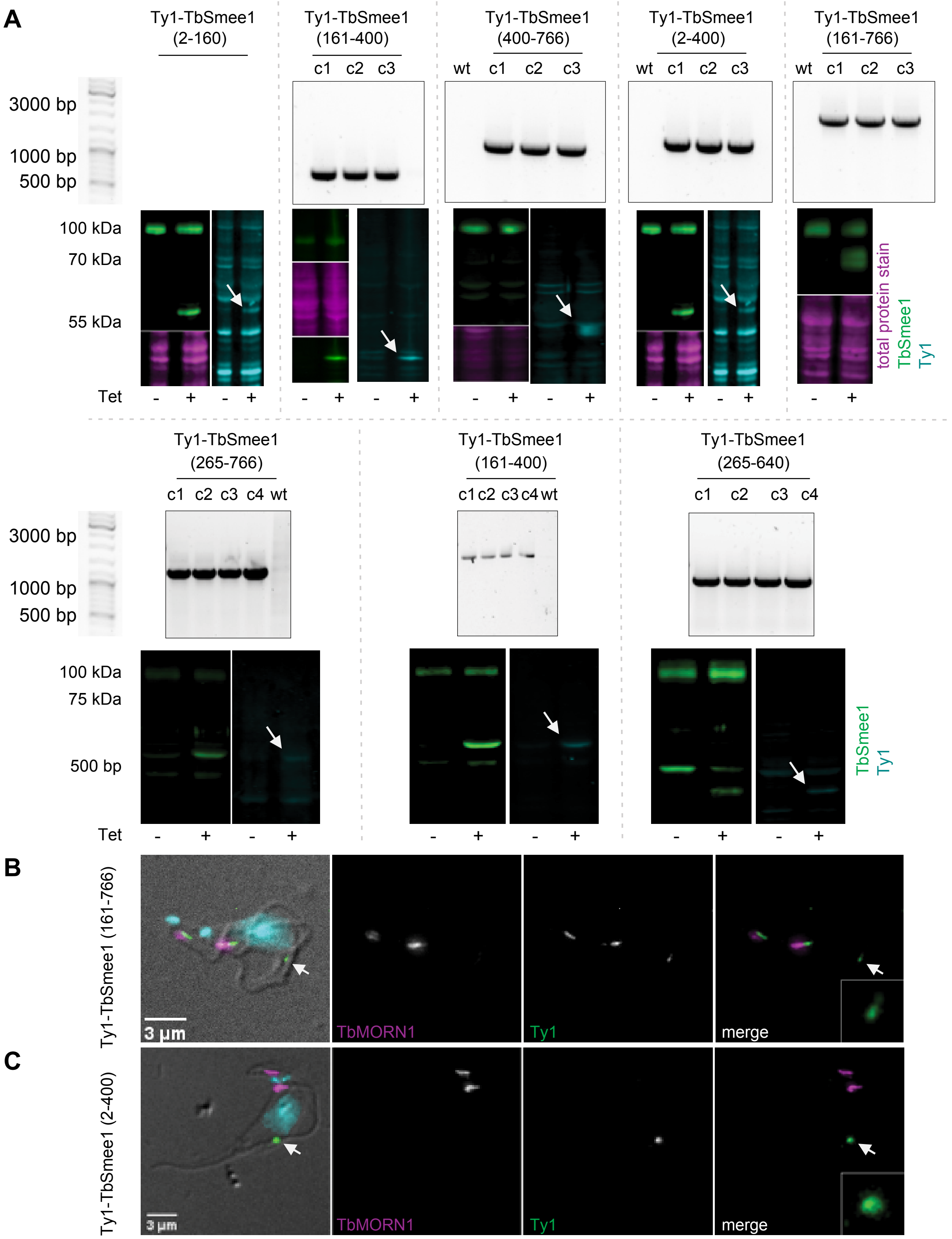

Journal of Cell Science • Supplementary information

Fig. S5. Validation of TbSmee1 truncations. (A) Validation of Ty1-TbSmee1 truncation cell lines by PCR analysis of genomic DNA and immunoblotting. The results for all 8 TbSmee1 truncations are shown, separated by dotted lines. Upper panels: PCR analysis of genomic DNA to confirm the presence of the truncation construct. PCR was used to amplify DNA from clones (c1, c2, c3) and wild-type control (wt) genomic DNA. Primers annealing to the sequence encoding Ty1 epitope and within the truncation were used. Lower panels: confirmation of Ty1-TbSmee1 truncation construct expression by immunoblotting. Whole-cell lysates from uninduced (-Tet) and induced (+Tet) cells were analysed by immunoblotting with anti-TbSmee1 (green) and anti-Ty1 (cyan) antibodies. Both antibodies detected proteins corresponding to the predicted size of the Ty1-TbSmee1 truncations; arrows indicate the target protein in the anti-Ty1 blots. A portion of the total protein staining of the membranes is shown in magenta. Note that the immunoblot for Ty1-TbSmee1(161-400) is the same data as shown in Figure 3C. (B) Ty1-TbSmee1(161-766) localises to both the hook complex and the FAZ tip. Cells expressing the Ty1-TbSmee1(161-766) construct were extracted with non-ionic detergent, fixed with methanol, and labelled with anti-TbMORN1 and anti-Ty1 antibodies. DNA was stained with DAPI (cyan). Ty1-TbSmee1(161-766) was observed at both the hook complex and the FAZ tip (arrow). (C) Ty1-TbSmee1(2-400) localises to the FAZ tip but not the hook complex. Cells expressing the Ty1-TbSmee1(2-400) construct were extracted, fixed, and labelled as above. Ty1-TbSmee1(2-400) was observed exclusively at the FAZ tip (arrow). Images in panels B and C are maximum intensity z-projections with a single DIC z-slice overlay. Multiple (n>2) independent experiments using three separate clones for each construct were carried out; exemplary cells are shown.

Journal of Cell Science • Supplementary information

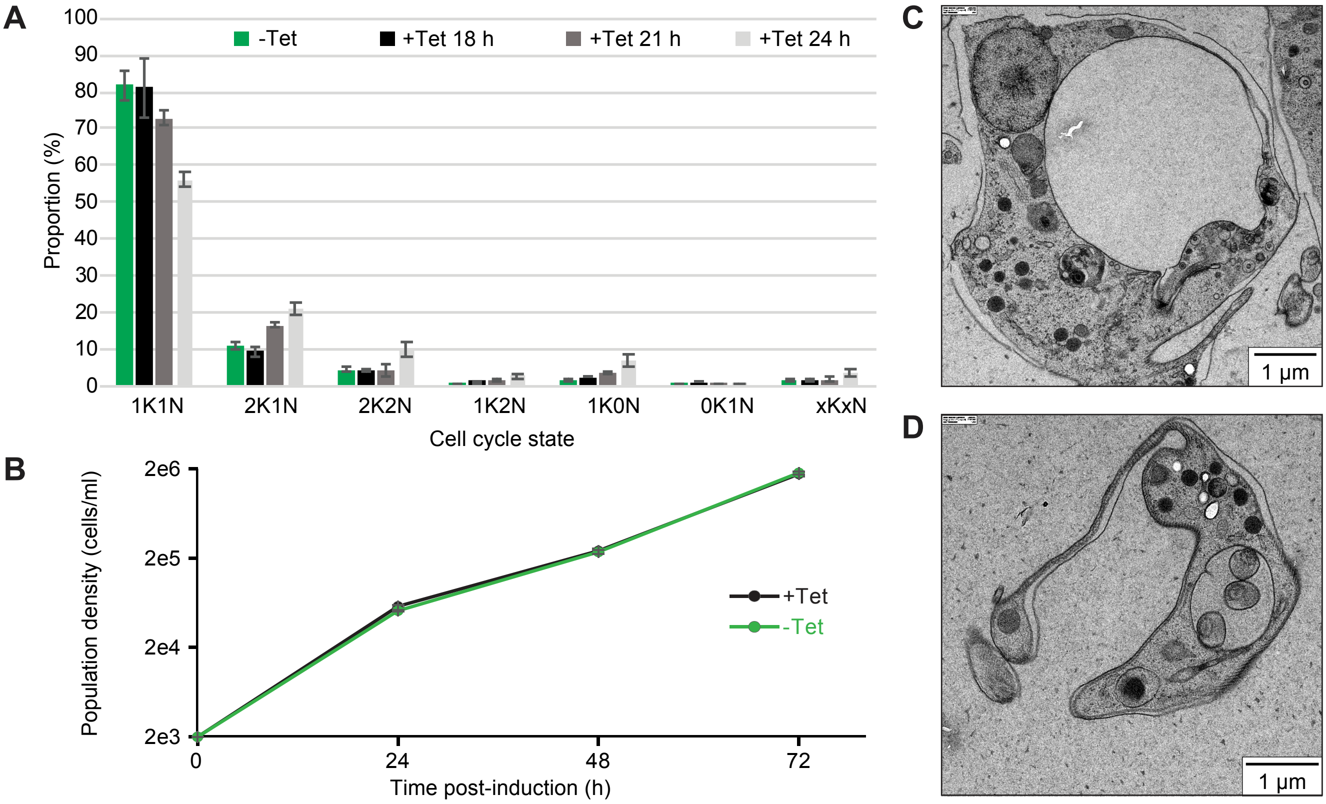

Fig. S6. Additional results for TbSmee1 and TbStarkey1 RNAi experiments. (A) TbSmee1 depletion causes changes to cell cycle state distribution. Control (-Tet) and TbSmee1-depleted (+Tet) RNAi cells from 18 h, 21 h, 24 h post-induction were fixed using glutaraldehyde; DNA was stained using DAPI. The various cell cycle states (1K1N, etc) were manually quantified from images taken of the fixed cells. Data were obtained from three independent experiments, each using three separate clones; at least 240 cells were quantified for each timepoint. (B) Depletion of TbStarkey1 has no effect on population cell growth. Control (-Tet) and TbStarkey1-depleted (+Tet) cells were followed over a 72 h timecourse, and population density (cells/ml) was measured every 24 h. Data were obtained from three independent experiments, each using 3 separate clones. (C, D) Depletion of TbStarkey1 causes morphological abnormality. TbStarkey1-depleted cells were prepared for electron microscopy using high-pressure freezing and imaged. Cells with enlarged flagellar pockets could readily be observed.

Journal of Cell Science • Supplementary information

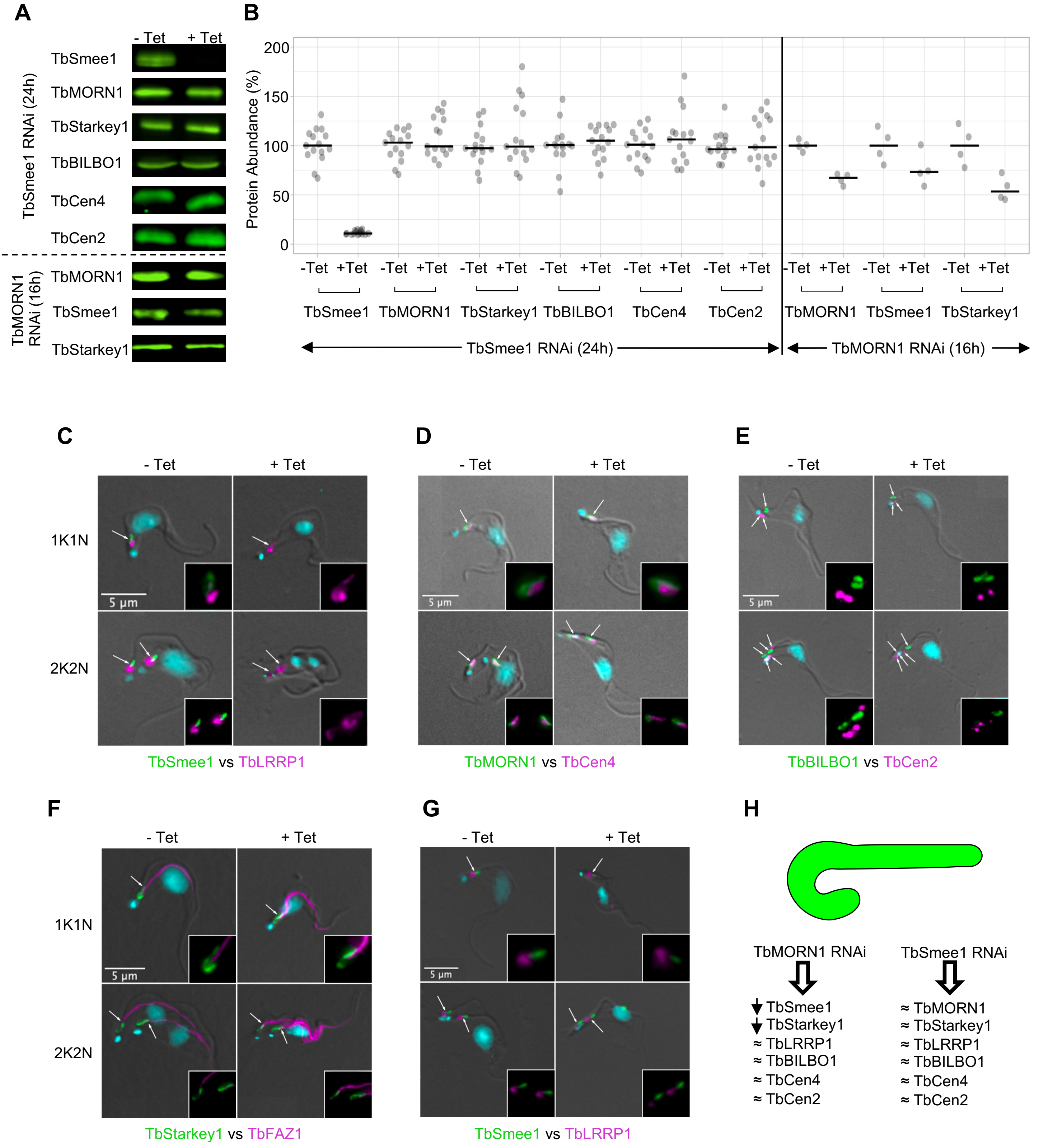
A
- Tet
+ Tet
TbSmee1
TbMORN1
TbStarkey1
TbBILBO1
TbCen4
TbCen2
TbSmee1 RNAi (24h)
TbMORN1
TbSmee1
TbStarkey1
TbMORN1 RNAi (16h)
B
Protein Abundance (%)
200
150
100
50
0
-Tet +Tet
TbSmee1
TbMORN1
TbStarkey1
TbBILBO1
TbCen4
TbCen2
TbMORN1
TbSmee1
TbStarkey1
TbSmee1 RNAi (24h)
TbMORN1 RNAi (16h)
C
- Tet
+ Tet
1K1N
2K2N
5 µm
TbSmee1 vs TbLRRP1
D
TbMORN1 vs TbCen4
E
TbBILBO1 vs TbCen2
F
TbStarkey1 vs TbFAZ1
G
TbSmee1 vs TbLRRP1
H
TbMORN1 RNAi
TbSmee1 RNAi
TbSmee1
TbStarkey1
≈ TbLRRP1
≈ TbBILBO1
≈ TbCen4
≈ TbCen2
≈ TbMORN1
≈ TbStarkey1
≈ TbLRRP1
≈ TbBILBO1
≈ TbCen4
≈ TbCen2

Fig. S7. Depletion of TbSmee1 affects other cytoskeleton-associated proteins. TbSmee1 RNAi cells were induced for 24 h and analysed by immunoblotting of whole-cell lysates and immunofluorescence imaging of detergent-extracted cells. (A) Exemplary immunoblots showing the effect of 24 h TbSmee1 depletion on marker proteins for the hook complex, flagellar pocket collar, and centrin arm. For comparison, the effect of 16 h TbMORN1 depletion was also analysed. (B) Quantification of immunoblot data. TbSmee1 depletion did not affect any of the proteins analysed. Total protein staining was used for signal normalisation. Normalised TbSmee1 signals in +Tet cells were expressed relative to the -Tet signal for each sample. TbSmee1 -Tet signals were expressed relative to the mean of all TbSmee1 -Tet values in the dataset. Immunoblots from TbMORN1 RNAi experiments were quantified in the same way; TbMORN1 depletion resulted in a loss of both TbSmee1 and TbStarkey1 signal. The data shown were obtained from multiple (n>2) independent experiments, each using three (TbSmee1 RNAi) or two (TbMORN1 RNAi) separate clones. (C-F) TbSmee1 depletion does not affect the localisation of the marker proteins. Control (-Tet) and TbSmee1-depleted (+Tet) cells were extracted with detergent, fixed with methanol, and labelled with the indicated antibodies; DNA was stained using DAPI. The position of the hook complexes in exemplary 1K1N and 2K2N cells are shown with arrows; panel E also indicates basal bodies. Data were obtained from 2 independent experiments for each labelling combination, each using 3 separate clones. (G) TbSmee1 and TbLRRP1 in TbMORN1-depleted cells.(H) Summary of the observed effects (arrow = depletion, squiggles = no change) on the abundance of marker proteins caused by either TbMORN1 or TbSmee1 depletion, based on immunoblotting and immunofluorescence data.

Journal of Cell Science • Supplementary information

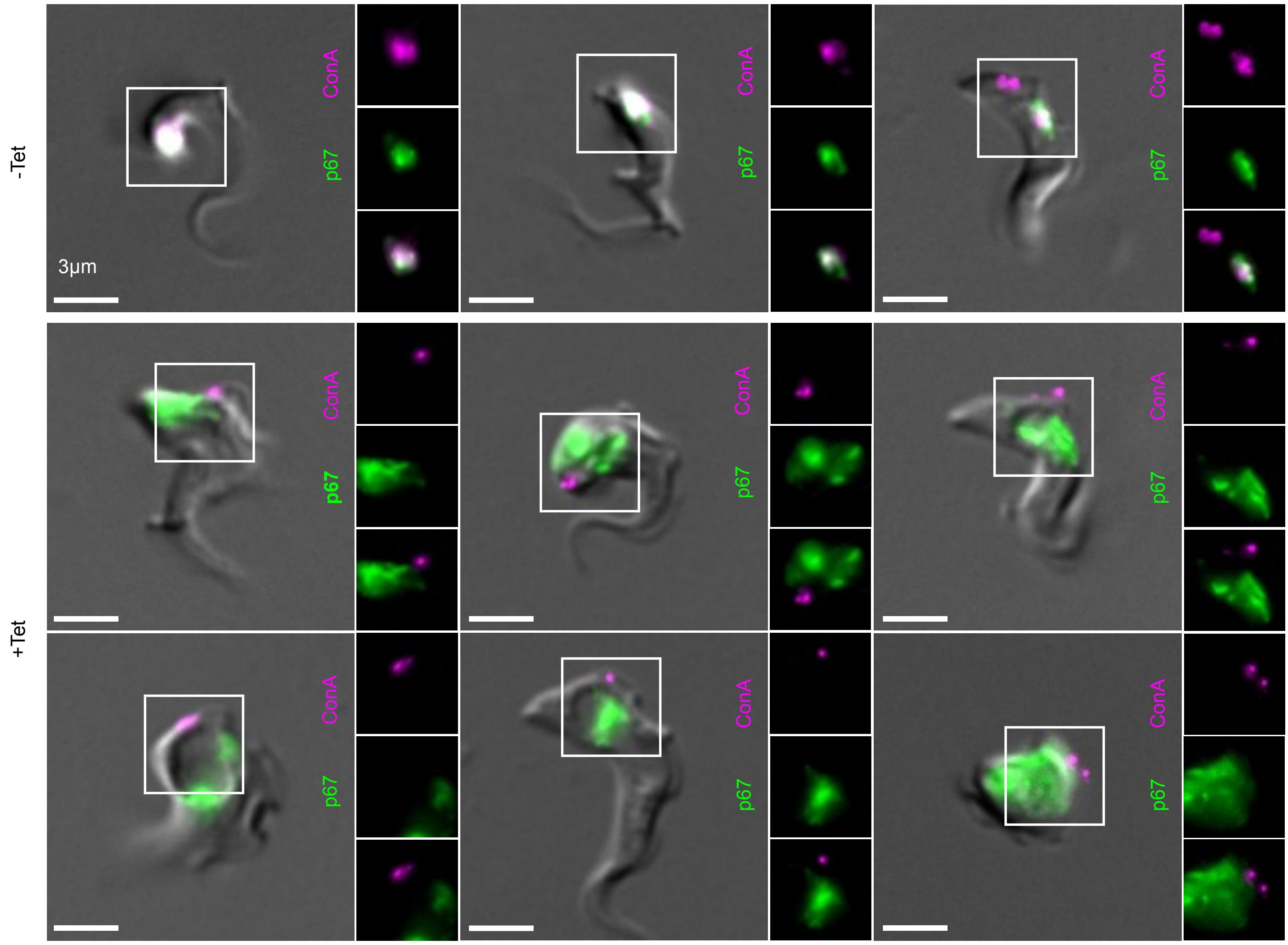

Fig. S8. Depletion of TbSmee1 prevents trafficking of ConA to the lysosome. Control (-Tet) and TbSmee1-depleted cells (+Tet; 24 h timepoint) were incubated on ice with ConA (magenta). The cells were then shifted to 37 ºC for 30 min to allow internalisation. The cells were then fixed and labelled with antibodies specific for the lysosome marker p67 (green). Control (-Tet) cells showed strong overlap between the two labels, indicating that ConA had been trafficked to the lysosome. +Tet cells showed no overlap between the two labels. Maximum intensity projections of the fluorescence channels are shown overlaid with a single DIC z-slice. Overlap in -Tet cells was confirmed in single z-slices. Single channels from the boxed area in each image are shown as insets. Data obtained from multiple (n>2) independent experiments each using three separate clones; exemplary cells are shown.

Supplementary reference

Corpet, F. (1988). Multiple sequence alignment with hierarchical clustering. *Nucleic Acids Res.* **16**, 10881-10890. doi:10.1093/nar/16.22.10881
