# Supplementary material for: Characterisation of TbSmee1 suggests endocytosis allows surface-bound cargo to enter the trypanosome flagellar pocket
Source: J Cell Sci. 2023 Oct 26;136(20):jcs261548. doi: 10.1242/jcs.261548 (PMC10652038; doi:10.1242/jcs.261548)
Supplement: Supplementary file 1 [file joces-136-261548_review_history.pdf]

Characterisation of TbSmee1 suggests endocytosis allows surface-bound cargo to enter the trypanosome flagellar pocket

Daja Schichler, Antonia Konle, Eva-Maria Spath, Sina Riegler, Alexandra Klein, Anna Seleznev, Sisco Jung, Timothy Wuppermann, Noah Wetterich, Alyssa Borges, Elisabeth Meyer-Natus, Katharina Havlicek, Sonia Pérez Cabrera, Korbinian Niedermüller, Sara Sajko, Maximilian Dohn, Xenia Malzer, Emily Riemer, Tuguldur Tumurbaatar, Kristina Djinovic-Carugo, Gang Dong, Christian J. Janzen, and Brooke Morriswood
DOI: 10.1242/jcs.261548

Editor: Michael Way

Review timeline

Submission to Review Commons:	16 March 2022
Submission to Journal of Cell Science:	10 August 2023
Accepted:	11 August 2023

<u>Reviewer 1</u>

Evidence, reproducibility and clarity

Unfortunately, this paper adds only a little to our understanding of uptake in to the flagellar pocket of trypanosomes. It tends to add only detail to information that has been well characterised elsewhere and indeed, as the authors themselves point out, (lines 92-98) it is rather incremental. Not only has Tbsmee1 been studied before but this data in bloodstream forms is not particularly novel since it gives much the same information as the canonical hook protein TbMORN.

This work follows the pattern of conclusions made previously with the protein TbMORN. It focusses on the protein TbSmee where RNAi mutants are interpreted to show flagellar pocket enlargement and impaired access by surface bound cargo. Unfortunately, there is little mechanistic or functional conclusion to the study in terms of how TbSmee operates naturally in the cell. There are other possible explanations for the phenotype. That would need to be studied. This large flagellar pocket phenotype is seen with RNAi mutants of many different types of proteins in the trypanosome and so pleiotropic effects are highly likely.

Also, there are a good number of alternative possibilities to account for reduced access to the pocket in these mutants and this data could be usefully added.

Specific points

1. The transient location for the TbSmee at the FAZ tip - or in this case the groove region - was seen in procyclics (Perry, 2018) so this bloodstream indication merely confirms that concept.
2. The C terminal region required for targeting is a reasonable deletion analysis of regions of the protein. But can this data (line 228) be said to "mediate targeting" - or is it just required. For instance, targeting might be OK but it might be needed for stable association, etc etc.
3. This protein has already been shown to be phosphorylated and the sites and cell cycle possibilities have been mapped by Urbaniak. So that section adds little. https://doi.org/10.1371/journal.ppat.1008129
4. Essentiality in BS forms and pocket enlargement. This is not surprising. A very large number of cytoskeletal proteins show this in RNAi knockdown. Flagella mutants (extensive

© 2023. Published by The Company of Biologists under the terms of the Creative Commons Attribution License (https://creativecommons.org/licenses/by/4.0/).

publications from many groups (Hill, Bastin, Gull, etc) over last 15 years show this very well and so this protein is just one more example.

5. I didn't find that the explanations for flagella pocket enlargement are soundly based. The experiments focus on endocytosis and uptake and ignore other plausible reasons and some evidence in literature.

Lines 84/85. Enlarged pockets may be indicative of endocytosis failure. Presumably the rationale is that endocytosis fails, but exocytosis still occurs and the pocket membrane enlarges. What evidence is there that exocytosis of membrane still occurs? This simple concept might indeed operate in a clathrin mutant but is surface membrane/content exocytosis is maintained in these cytoskeleton mutants? There is good evidence for glycoconjugates within the flagellar pocket. Are these depleted or present still?

6. There are also a number of other publications indicating that clathrin pits are still present on the enlarged pockets of various mutants when viewed by EM. The authors have looked at the flagellar pockets by EM but the EM methods described have extensive washings and centrifugations before fixation. This is a very poor approach and will mean that endo and exocytic traffic is disturbed (extensive references in literature in other systems? This is not a useful approach for exo/endocytosos studies where flux of traffic demands fast chemical or freezing fix in media.

7. The EMs and Light microscopy does show that the mutant pockets are substantially abnormal in their cytoskeletal arrangement. They have multiple flagella profiles, flagella structures have not connected with the membrane and are sometimes in the cytoplasm (see a glance of the paraflagellar rod in the cytoplasm in FigS5C and internalised FAZ attachment plaques in Fig 4 D bottom right cell). Given these extensive (and expected) cytoskeletal abnormalities it is highly likely that these pocket abnormalities are a result of motility, cell division/developmental issues and the differential uptake phenotypes merely consequential.

8. The authors speak about early phenotypes , but these are often at 15-24 hours. That is probably a couple of cell cycles and so not early. In relation to the above question of comparison to the same morphology produced by flagella mutants it would be good to know if these hook mutants produce motility phenotypes and whether these are manifest before the uptake phenotypes. There is evidence (cited here) that forward motility of the trypanosome directs material on surface into the pocket. If these cells have motility defects (primary or via failed division) then surely that would provide an alternative simple explanation for uptake differences.

There is a general point that if studies are to have real relevance to uptake in the trypanosome then they need to deal with uptake of natural ligands rather than artificial surrogates such as dextran. Such tracers were used historically, but in the last decade a series of receptors and ligands for fluid phase and particularly membrane mediated endocytosis have been discovered. With the investment of a little time these important ligand / receptors such as haptoglobin, transferrin, etc would be much more relevant.

Significance

Unfortunately, I did not find tis to be very significant. It covers old ground in terms of the phenotype described. Many groups have shown the differences between pro cyclic and bloodstream phenotypes in this enlarged pocket phenomenon. The work is rather incremental from these and other author's work on these hook proteins.

There are alternative explanations for understanding the effect of flagella pocket structure and uptake of ligands into the pocket and trypanosome cell. These would need to be studied before one could see a functional, mechanistic link established.

Other parts of this are of nicely done but do not move on our understanding (eg targeting/phosphorylation) from what has been done previously.

Referees cross-commenting

This session includes comments from Reviewer 1 and Reviewer 2.

© 2023. Published by The Company of Biologists under the terms of the Creative Commons Attribution License (https://creativecommons.org/licenses/by/4.0/).

Reviewer 2

Dear Reviewers 1 and 3:
I agree with many of the points with Reviewer 1 and our divergence is partly a matter of degree. While it is true that this manuscript is incremental in its contribution to our understanding of TbSmee1, it nonetheless adds to our understanding of the role of this protein in the bloodstream life stage and because of that I find value in the work. The fact that it mirrors what was seem in other protein knockdown studies (e.g. TbMORN) doesn't negate its contribution for me. Reviewer 1 makes an important point, however, when stating that this work does not add a mechanistic or functional conclusion as to how TbSmee1 operates and for me that is the biggest shortcoming of the work. Offering mechanistic insight is a high bar and while it would make for a much more exciting story it does not discount the value of the work as presented. What I do appreciate is the speculation about this observation that endocytosis is required for entrance of surface bound material into the pocket and although they are unable to show that this is not a side affect of other processes being disrupted it is and intriguing point. These observation have the potential of stimulating further investigations into crosstalk between the entrance to the pocket and endocytosis. I also agree that the use of ligands for known receptors like transferrin would be far more informative. While I assumed the transferrin receptor was in the pocket itself it would be interesting to see if the ESAG6/7 is also located outside the pocket and transiently binds cargo before being brought inside for endocytosis.

I think that Reviewer 3 brings up a great point with the focus on VSG's. I think that examining VSG turnover in these mutants can add value to the analysis and inform our view of how affecting the hook complex alters VSG endocytosis.

Reviewer 1

Some fair comment and agreement. This is being sent to general cell biology journals. when one looks at this area in the round it is nearly 50 years (1975) since Langreth and Balber published their seminal work on protein uptake and digestion in bloodstream and culture forms of T. brucei. There has been 50 years intense study and the genome has been around for nearly 20 years as well. So, put simply - for both a general science audience and the wider parasite community - if this is a paper about one protein, TbSmee1,then it has surely has to say something functional about that protein. If it is a paper about uptake in trypanosomes (where mutants are one means of interrogation) then it surely has to say something about mechanisms of uptake of physiological relevant ligands. The days of dextran etc are past. Hence, my comment that this does neither and so is very incremental to what is known already. It is 2022 not 1975. Langreth and Baber published their seminal work in J Protozoology for very good reasons no doubt.

Reviewer 2
Thank you for replying and I agree with the spirit of your critique. My only comment, which could result from my own naivete, is to say that despite the incredible work that has been done in dissecting endocytosis in T. brucei over these past 50 years, it appears that we still do not understand how many fundamental of aspects of this activity works in this parasite. Even basic questions regarding how cargo, e.g. transferrin, binding to surface receptors is sensed by the parasite remains unknown and the identity of the specific signaling components which transmit this information internally to initiate endocytosis have not been characterized. In many ways it seems that we don't even understand how the parasite partitions the end/exocytic pathways in the pocket and maintains membrane homeostasis. While we know that some kinases and traditional signaling components must be involved, a high resolution understanding of this process in T. brucei seems lacking. I only say all this to suggest that the field maybe isn't yet that advanced to reject work of this type as so many mechanistic unknowns still remain to be uncovered and maybe incremental advances and phenomenology still can add value to the field. However, I respect your opinion on the matter and my perspective could be due to a lack of a full appreciation of the literature on the subject.

© 2023. Published by The Company of Biologists under the terms of the Creative Commons Attribution License (https://creativecommons.org/licenses/by/4.0/).

Reviewer 2

Evidence, reproducibility and clarity

Summary:

In this manuscript the authors have advanced our understanding of the hook complex component TbSmee1 through a detailed analysis of this protein's role in the endocytosis of surface bound proteins via the flagellar pocket in bloodstream form Trypanosoma brucei. The TbSmee1 protein, previously identified using proximity labeling using TbMORN1 and TbPLK, and characterized in procyclic T. brucei, was confirmed to target to both the shank portion of the hook complex as well as the growing end of the new FAZ in replicating cells. The protein was also shown to likely be phosphorylated as had been suggested previously due to its association with the kinase TbPLK. A domain deletion analysis demonstrated that domains 2 and 3 are important for TbSmee1's proper localization to the hook complex. Loss of TbSmee1 using RNAi based knockdown resulted in a quick cessation of growth in the bloodstream form within 24 hours in contrast to what was seen previously in procyclic cells which had only a decreased growth rate. Loss of TbSmee1 also resulted in an enlargement of the flagellar pocket and in many ways mirrored the phenotype observed with knockdown of TbMORN1. Although prior work on TbSmee1 in procyclic T. brucei demonstrated that loss of this protein altered the morphology of TbMORN1, no such change was seen in bloodstream form cells and only an alteration in the morphology of TbLRRP1 was observed. In characterizing the effect of TbSmee1 depletion on endocytosis the authors showed that the fluid phase marker Dextran could enter into the flagellar pocket of TbSmee1 depleted parasites while the surface bound ConA and BSA remained outside of the flagellar pocket suggesting that TbSmee1 may play a role in allowing larger protein components into the pocket regions. Similar observations were also previously seen with TbMORN1 depletion. Importantly, a knockdown of clathrin recapitulated the TbSmee1 knockdown phenotype suggesting that endocytosis itself was required to allow material bound at the surface to enter into the flagellar pocket. In addition to adding to our understanding of hook complex components, this work raises some interesting questions regarding the role of the hook complex in facilitating endocytosis in this important human pathogen.

Major Critiques:

This is a superbly written manuscript with robust high-quality data that strongly support the major conclusions made by the authors. The flow the article is logical and easy to follow making it accessible to a wide array of readers. Although I appreciate the brevity of the introduction and how the article gets straight to the point, additional background information on the components and function of the flagellar pocket collar protein could help contextualize the goals of the project. The way in which the flagellar collar structures are introduced to the reader is quite abrupt (beginning on line 75) and simply states the names of TbBILBO1, the centrin arm and hook complex as simple facts without much discussion about the background of these components/regions. A graphical representation of the centrin arm or hook complexes relative to other components like the pocket itself, FAZ or axoneme could make following the story much easier. An expansion of this background could also go a long way to convince readers of the importance of this region in the basic biology and virulence of T. brucei.

On lines 84-86 the authors cite the way in which 'small' vs 'large' macromolecules enter into the pocket without defining what exactly is meant by these terms as they are relative in nature. Setting some boundaries of size could provide some context to the reader.

In the domain localization analysis beginning in Figure 4 there is a missed opportunity to also assess which portions of the TbSmee1 protein are important for overall function as well. By either an examination of dominant negative phenotypes resulting from overexpression of the truncated mutant or the expression of the truncated forms designed to be RNAi resistant in the TbSmee1 knockdown cell line, one could also assess which portions of this protein are essential for endocytic function in addition to targeting. Is there a reason this was not performed?

In the analysis of viability changes due to TbSmee1 depletion (lines 237) the authors state that at "72 h post-induction showed widespread lysis, ..." This phenotype seems inconsistent with other

© 2023. Published by The Company of Biologists under the terms of the Creative Commons Attribution License (https://creativecommons.org/licenses/by/4.0/).

related endocytic defect mutants. There is no further mention of this lysis phenomenon here or in the discussion and considering how unique this seems it deserves either additional data to demonstrate or further discussion as to the basis of the phenotype. It seems, at least from this study of TbStarkey1 and prior studies which result in the enlarged flagellar pocket phenotype, that having an enlarged pocket is not the cause of lysis and doesn't even naturally lead to a growth defect.

The authors do not comment on what is the source for the cessation in growth following TbSmee1 knockdown. Is it nutrient depravation like in other endocytic defect mutants?

In the end, one of the most interesting observations made by the authors is that loss of TbSmee1 inhibits endocytosis and this has the appearance of not allowing large molecule substrates like ConA and BSA to enter into the flagellar pocket. This appeared to have nothing to do with a gatekeeping type function of the hook complex/flagellar collar and instead, as shown through clathrin knockdown, was related to the ability of the parasite to endocytose. There are a lot of potential interpretations of this phenomenon with one being a simple perturbation of the normal membrane trafficking to and from the flagellar pocket being involved. An analysis of knockdown of exocytic components might reveal whether or not this inability to enter into the pocket is also seen when exocyst proteins are also depleted. It may be impossible to tease apart these two interrelated activities but it might eliminate one side of the equation if these proteins can still enter the flagellar pocket when exocytosis if perturbed although this reviewer understands that that dimension of T. brucei membrane trafficking is poorly understood relative to endocytosis.

An intriguing possibility that the authors allude to and which if answered would make this manuscript have a far broader appeal is to determine if loss of TbSmee1 alters the lipid kinase distribution and if this is the source of the negative impact on endocytosis. One important dimension of endocytosis in T. brucei which remains poorly understood is the role of signaling machinery in triggering endocytic events. It is possible that the hook complex serves as the gatekeeping or signaling platform that recruits signaling components (like lipid kinases) that identify and/or modify the membrane lipid phosphatidylinositols harboring cargo laden receptors thus marking them for endocytosis within the pocket. It still seems unclear when in the process of endocytosis is the decision made to pull things into the pocket but it seems that the assumption is that this occurs deep within the pocket. This data suggests that there is possibly another decision point prior to being allowed entrance into the pocket. It may be that this isn't a gatekeeping decision but rather a stop vs. go activity where once cargo laden membrane reaches the collar a choice is made to pull this material in or not there and not after material is already in the pocket.

This obvious enigma based on the observation that loss of hook complex components affect the spatially separated site of endocytosis support the idea that the actual endocytic signaling platforms are located at the hook complex and that this area may make the membrane modifications that mark membrane as being ready to be endocytosed via clathin coated vesicles at the bottom of the pocket. This would still allow for fluid phase small molecule entrance which does not require binding to surface proteins. The obvious problems of having both endo/exocytosis occurring in the same close proximity makes the dissection of this phenomenon difficult but it is worth potentially expounding on further in the discussion as this idea is very appealing and adds an important dimension to our understanding of endocytosis in this organism.

Minor Critiques:

The authors commit significant time to the analysis of the phosphorylation of TbSmee1, but there is little stated about the role of TbPLK in this activity or the potential connection of TbSmee1 phosphorylation to the cell cycle. Would a knockdown of TbPLK using RNAi potentially demonstrate an altered migration of TbSmee1 due to a lack of phosphorylation? An analysis of radiolabeled TbSmee1 using p32 in vivo would likely support this claim as well. Has mass spectrometry identified potential phosphorylation sites to examine?

Additionally, the loss of TbSmee1 has been shown to disrupt localization of TbPLK in procyclic cells and so why this was not also assessed in bloodstream form cells subjected to RNAi was not clear.

© 2023. Published by The Company of Biologists under the terms of the Creative Commons Attribution License (https://creativecommons.org/licenses/by/4.0/).

In the results section (lines 104-108) a model of the protein structure as predicted for example by AlphaFold might be informative and complement the domain analysis work depending on the quality of the prediction.

There is an arrow in the Figure 1B Western blot but I can find no mention of what it is trying to highlight in the text.

For Figure 1D there is no loading control or control for the distribution of the soluble fraction to validate the separation of the two compartments.

The authors fail to comment on the lack of changes in hook complex components they see to that observed by Perry et. al. 2018. This difference merits some minor comment or speculation.

Line 228: domain should be capitalized.

Line 230: FigS5C should have a space and period after Fig. and S5C.

Line 244: "on" should be inserted in the sentence "...TbSmee1 protein depletion ON either side of the onset..."

Line 400: the '...20/21 h post-induction...' is slightly confusing and may read better as 20-21 h.

Line 463: a space is needed between '...2009).The...'.

Significance

This manuscript advances our current conception of endocytosis in T. brucei. Although this model kinetoplastid parasite has been extensively studied with respect to endocytosis there is still a great deal we do not yet understand regarding how this process is regulated at a mechanistic level. This work has begun to connect previously unappreciated aspects of endocytosis in T. brucei by highlighting a potentially novel connection between the flagellar collar/hook complex and the physically separated endocytic events within the flagellar pocket itself. It may be that what appears as regulated entrance into the pocket is in fact the source of signaling that triggers the endocytic events carried out by clathrin. This is an interesting notion that no doubt requires further investigation which lies outside of the scope of this report. While this work appeals primarily to those studying kinetoplastids parasites it has the potential to provide insight into basic protozoan biology as well. Due to my related interest in kinetoplastid endocytosis, I find this work to be of high quality, conceptually interesting and employs many of the cutting-edge techniques currently available in the study of T. brucei.

Reviewer 3

Evidence, reproducibility and clarity

This manuscript begins to dissect the function of the hook complex protein SMEE1 in the mammalian infective form of T. brucei. The hook complex is a cytoskeletal structure associated with the flagellar pocket, the only site of endo/exocytosis in these cells. The authors demonstrate that SMEE1 is required for endocytosis in these cells and that this can occur with minimal change to the molecular make-up of the hook complex. The authors show that endocytosis is important for the access of large molecules e.g. ConA into the flagellar pocket.

Major comments

The key conclusion of this study are convincing and the data is generally well presented and clear. The interpretation of the figures matches well with the data presented - there are a few minor issues though that I have highlighted below in minor comments. The authors use a range of

© 2023. Published by The Company of Biologists under the terms of the Creative Commons Attribution License (https://creativecommons.org/licenses/by/4.0/).

molecular cell biology approaches to define the role of SMEE1 and these are appropriate and are well controlled.

My major comment focuses on the use of different tracers to study endocytosis but the elephant in the room is what is happening to VSG as this is the surface protein that needs to rapidly removed from the cell surface and cleaned. Given the importance of removal of antibodies bound to the VSG - have the authors looked at this in the SMEE1 depleted cells? Do VSG-antibody complexes accumulate in this region? This is an important experiment as this would give key physiologically relevant data to this study. All the material should be readily available for this as there are a number of VSG antibodies.

Minor comments

Perhaps I have been overthinking this but is surface-bound the right way to describe the cargo, as it clearly goes in both directions onto and off the surface and in fact the experiments in this manuscript are focussing on the removal of this material from the surface so is not surface-bound.

Have the authors investigated the structure of the protein using alphafold and if so how does that compare to the domain structure that was presented in this manuscript?

The authors raised a number of antibodies to TbSMEE1 and TbSTARKEY1 but it was not clear in the figures which antibody was ultimately used for analysis by western and IF - could the authors clarify, as some looked to have a higher background than others. Line 150 states the same localisation was seen for all three antibodies and references S3C but I couldn't see that data presented.

Line 169 - can the authors provide more detail about the global correlation methodology as I was unable to follow the details in the methods? Is this a pixel per pixel correlation over the image or on a selected region over the area of potential signal overlap? In figure 2E it appears that BILBO1 signal correlates more closely with the SMEE1 signal than MORN and LRRP1 and from the images that would not seem to be the case. Have I interpreted this figure incorrectly?

The authors have generated a number of different clones and performed experiments on these clones generally more than twice, which is clearly explained in the figure legends but in places the data is then put together and it is difficult to know which experiments/clones it comes from - for example 7C/7F what do those percentages represent? Is this the sum of all experiments? A representative experiment? How many cells per experiment were analysed?

Line 200 - From the image it is not convincing that SMEE1 is slightly behind DOT1 - I agree it looks enveloped but would appear level with the distal end of the DOT1 signal.

For the truncation experiments the authors should explain that these are performed with cells in which the endogenous SMEE1 will be expressed and this may influence the localisation of the truncations, especially as there is no information about whether SMEE1 forms complexes with itself or other proteins.

Figure 4D - should be 1 not T-

Line 223 - given the low expression of constructs 2 and 9 I'm not sure it is possible to infer anything from the lack of localisation of these constructs as they appear unstable and would be unlikely to localise to a specific location.

Figure S7 - The images presented were not convincing that there was a reduction in the localisation of LRRP1 to the hook complex on depletion of either SMEE1 or MORN1. The difference looks particularly minor if present at all.

Line 264 - "implied that the lethal phenotype might be due to a loss of function" - this seems an odd thing to say as it doesn't provide any insight as of course the phenotype is due to a loss of function.

© 2023. Published by The Company of Biologists under the terms of the Creative Commons Attribution License (https://creativecommons.org/licenses/by/4.0/).

Line 412 - can the authors clarify what they mean by geometric problems?

Throughout the manuscript can you use log scale for the growth curves.

Line 756 - add citation

Line 465/66 - the authors states that the ability of the fluid phase cargo being still able to enter the pocket is evidence that the channel lumen is still open; however, I would think that despite the close apposition of the cell membrane to the flagellar membrane in the flagellar pocket neck region this would be unlikely to impede fluid/soluble material from entering the pocket, as presumably VSG protein can move through this region. This does not alter the ultimate conclusion the authors are drawing but without microscopy evidence for the state of the channel lumen it is difficult to be sure of its status.

Significance

The flagellar pocket is the key portal into and out of the trypanosome cell and as such has a vital role to play in host-parasite interactions. The flagellar pocket is supported by a number of cytoskeletal structures including the hook complex and the role of these structures in flagellar pocket function are poorly understood. The flagellar pocket is particularly important in the bloodstream form of the trypanosome parasite which infects the mammalian host as it is the route for the surface protein VSG to get onto and off the surface. The VSG is required for antigenic variation and the removal of VSG-antibody complexes helps 'clean' the surface of the parasite. SMEE1 is a component of the hook complex and the manuscript here dissects its role in the mammalian infective parasite and shows that it is vital for the endocytosis of material off the surface. Intriguingly, a block in endocytosis causes a blockage of material outside of the pocket, suggesting a multi-step process in the regulation of uptake of material from the parasite's surface.

This manuscript will be of specific interest to those researchers investigating the long-term persistence of these parasites in the mammalian host. There are potentially some insights into the control of membrane domains for endocytosis that are of interest to more general cell biologists as well.

Expert in molecular cell biology of trypanosomes and Leishmania.

Author response to reviewers' comments

Reviewer #1 (Evidence, reproducibility and clarity (Required)):

Unfortunately, this paper adds only a little to our understanding of uptake in to the flagellar pocket of trypanosomes. It tends to add only detail to information that has been well characterised elsewhere and indeed, as the authors themselves point out, (lines 92-98) it is rather incremental. **We were disappointed that the reviewer was so unsupportive of the work presented here. It seems possible that the reviewer is partly objecting that the title - which emphasised the main finding of the paper - does not fully capture the content of the paper. We have therefore modified the title to emphasise that the paper is principally a characterisation of TbSmee1 rather than an investigation of the flagellar pocket, with the insight into cargo entry being the most notable finding.**

Not only has Tbsmee1 been studied before but this data in bloodstream forms is not particularly novel since it gives much the same information as the canonical hook protein TbMORN. This work follows the pattern of conclusions made previously with the protein TbMORN. It focusses on the protein TbSmee where RNAi mutants are interpreted to show flagellar pocket enlargement and

© 2023. Published by The Company of Biologists under the terms of the Creative Commons Attribution License (https://creativecommons.org/licenses/by/4.0/).

impaired access by surface bound cargo. Unfortunately, there is little mechanistic or functional conclusion to the study in terms of how TbSmee operates naturally in the cell.
This is deliberately downplaying the value of the work. TbSmee1 has not previously been characterised in bloodstream form cells, and neither TbMORN1 nor the hook complex are as well-characterised as other cytoskeletal components such as the flagellum and basal body. To criticise the paper for not providing a molecular mechanism of TbSmee1's function is unreasonable given the volume of work provided and the fact that this is a first characterisation of the protein in this life cycle stage. Expectation of a complete molecular mechanism is setting a very high bar for a first characterisation.

It is also possible that the reviewer has not grasped the main thrust of the argument - when TbMORN1 was characterised it was the first protein shown to have this cargo entry defect. We show here that not only does TbSmee1 share this defect, but that it is in fact a previously-unacknowledged feature of all phenotypes of this type, exemplified by clathrin. We have modified the text to make this finding more clearly emphasised (see for example lines 654-661 in the tracked-changes version of the manuscript).

There are other possible explanations for the phenotype. That would need to be studied. This large flagellar pocket phenotype is seen with RNAi mutants of many different types of proteins in the trypanosome and so pleiotropic effects are highly likely. Also, there are a good number of alternative possibilities to account for reduced access to the pocket in these mutants and this data could be usefully added.

This is another statement that seems intended primarily to disparage the paper rather than attempt to improve it. It would have been extremely helpful if the reviewer highlighted what these other possible explanations are instead of making vague allusions. The widespread prevalence of this kind of phenotype means that our insight into restricted cargo access to the flagellar pocket is of general relevance in the trypanosome field.

Specific points
1. The transient location for the TbSmee at the FAZ tip - or in this case the groove region - was seen in procyclics (Perry, 2018) so this bloodstream indication merely confirms that concept.
The reviewer is again downplaying the value of the work rather than providing constructive criticism. While FLAM3 has been shown to be at the tip of the new flagellum in bloodstream form cells (Sunter et al., 2015), at the time of the preprint being published Smee1 was actually the first protein (besides the DOT1 antigen) shown to localise to the groove region in bloodstream form cells. It is also worth noting that procyclic form cells and bloodstream form cells are fairly different in this regard - in procyclic cells, there is an entire flagellar connector structure that is not present in bloodstream form cells, and so demonstrating that Smee1 was present in the groove region was an important experiment. Since this preprint was published, Smithson et al. have identified 13 additional proteins localising to the groove (Smithson et al., 2022) - we have modified the text to include these points (see lines 542-545 of the tracked- changes manuscript).

2. The C terminal region required for targeting is a reasonable deletion analysis of regions of the protein. But can this data (line 228) be said to "mediate targeting" - or is it just required. For instance, targeting might be OK but it might be needed for stable association, etc etc.
We have changed the text to say "required" for targeting instead of "mediating" targeting (line 312 of tracked-changes manuscript).

3. This protein has already been shown to be phosphorylated and the sites and cell cycle possibilities have been mapped by Urbaniak. So that section adds little.
https://doi.org/10.1371/journal.ppat.1008129
The reviewer is again disparaging the significance of the work rather than critiquing it. This is after all only a single panel of a figure and ~15 lines of text, and therefore a minor but still noteworthy element of the manuscript. This also misunderstands what the Urbaniak study does and does not show - while that work showed that Smee1 is phosphorylated, it remained possible that other post-translational modifications were occurring. This experiment shows that the "fuzzy" appearance (variable electrophoretic migration) of TbSmee1 in gels can be solely attributed to phosphorylation as opposed to other post-translational modification. We contacted Dr. Urbaniak to confirm this - his

© 2023. Published by The Company of Biologists under the terms of the Creative Commons Attribution License (https://creativecommons.org/licenses/by/4.0/).

answer is below.

"I think your approach to look at the fuzzy banding is actually rather elegant; our data shows that phosphorylation occurs but we did not look for any other PTMs that could influence migration on a gel and probably wouldn't see them without a different enrichment and analysis method. We often see a fuzzy pattern with glycosylation due to the heterogeneity, and I suspect other modifications will also results in a smear. Given that the band collapses to a single band after phosphatase treatment and not with an inhibitor present it is fair to conclude that phosphorylation is responsible for the fuzzy band, not other undefined PTMs like glycosylation."

4. Essentiality in BS forms and pocket enlargement. This is not surprising. A very large number of cytoskeletal proteins show this in RNAi knockdown. Flagella mutants (extensive publications from many groups (Hill, Bastin, Gull, etc) over last 15 years show this very well and so this protein is just one more example.
This appears to be another comment aimed at downplaying the value of the manuscript rather than providing constructive feedback. The fact that we have demonstrated something previously unobserved in a common phenotype makes the data of general interest to the community, we feel.

5. I didn't find that the explanations for flagella pocket enlargement are soundly based. The experiments focus on endocytosis and uptake and ignore other plausible reasons and some evidence in literature.
Again, the reviewer's feedback would be considerably more constructive if they had taken the time to specifically cite the evidence in the literature that they are alluding to, and present some of the "other plausible reasons" they are aware of. We have consulted widely in the community and have not been able to find anybody who knew what work the reviewer is referring to here.

Lines 84/85. Enlarged pockets may be indicative of endocytosis failure. Presumably the rationale is that endocytosis fails, but exocytosis still occurs and the pocket membrane enlarges. What evidence is there that exocytosis of membrane still occurs? This simple concept might indeed operate in a clathrin mutant but is surface membrane/content exocytosis is maintained in these cytoskeleton mutants? There is good evidence for glycoconjugates within the flagellar pocket. Are these depleted or present still?
The reviewer is correct that we have not specifically assayed for exocytosis, but the fact that we are able to make the same observations in both the clathrin RNAi (where exocytosis has been assayed - Allen et al., 2003) and the Smee1 RNAi means that this is not a problematic omission. The effect of the enlarged flagellar pocket phenotype on the glycoconjugates in the flagellar pocket is an interesting question but far outside the current focus of the paper.

6. There are also a number of other publications indicating that clathrin pits are still present on the enlarged pockets of various mutants when viewed by EM. The authors have looked at the flagellar pockets by EM but the EM methods described have extensive washings and centrifugations before fixation. This is a very poor approach and will mean that endo and exocytic traffic is disturbed (extensive references in literature in other systems? This is not a useful approach for exo/endocytosos studies where flux of traffic demands fast chemical or freezing fix in media.
The reviewer has misunderstood the aim of the experiments described in Figure 5D, which was to observe the morphological changes caused by depletion of TbSmee1. As the reviewer is no doubt aware, high-pressure freezing of trypanosomes gives much better morphological preservation than chemical fixing in media, so the choice of method is not "very poor" but tailored to the experimental aims. We have modified the text to make this point more clearly (lines 355-358 of tracked-changes version). Once again, the referee offers no citation to back up their assertion that endo- and exocytic traffic is disturbed by wash steps, either in trypanosomes or elsewhere.

7. The EMs and Light microscopy does show that the mutant pockets are substantially abnormal in their cytoskeletal arrangement. They have multiple flagella profiles, flagella structures have not connected with the membrane and are sometimes in the cytoplasm (see a glance of the paraflagellar rod in the cytoplasm in FigS5C and internalised FAZ attachment plaques in Fig 4 D bottom right cell). Given these extensive (and expected) cytoskeletal abnormalities it is highly likely that these pocket abnormalities are a result of motility, cell division/developmental issues and the differential uptake phenotypes merely consequential.
This is another misinformed argument that is seeking to disparage the data. The reviewer has

© 2023. Published by The Company of Biologists under the terms of the Creative Commons Attribution License (https://creativecommons.org/licenses/by/4.0/).

apparently overlooked the fact that the same phenotype is seen in clathrin RNAi, when flagellar pocket enlargement precedes any downstream effects on cell division cycle progression. We have gone to great lengths (Fig 6) to demonstrate that the enlargement of the flagellar pocket almost certainly precedes the onset of the growth defect in the TbSmee1 RNAi, and it is therefore likely to precede the cytoskeletal abnormalities that the reviewer has highlighted. An effect on cellular motility is possible and would be interesting to investigate in future work.

8. The authors speak about early phenotypes , but these are often at 15-24 hours. That is probably a couple of cell cycles and so not early.
To be informative, the analyses of RNAi phenotypes have to be done as soon as possible after the onset of the growth defect, and we have gone to great lengths (Figure 5) to define this point as being at 21 hours. This is already difficult as the number of phenotypic cells at the onset of the growth defect will not be high. We have clarified the text to emphasise that "early" refers to soon after the onset of the phenotype (lines 388-389 of tracked-changes version).

In relation to the above question of comparison to the same morphology produced by flagella mutants it would be good to know if these hook mutants produce motility phenotypes and whether these are manifest before the uptake phenotypes. There is evidence (cited here) that forward motility of the trypanosome directs material on surface into the pocket. If these cells have motility defects (primary or via failed division) then surely that would provide an alternative simple explanation for uptake differences.
The reviewer is overlooking the observation that the surface-bound endocytic cargoes (ConA, BSA) are still being sorted/directed as far as the entrance to the flagellar pocket - what is interesting is that the cargo is apparently unable to enter the flagellar pocket. As noted above, it would certainly be interesting to look at motility effects in follow-up work.

9. There is a general point that if studies are to have real relevance to uptake in the trypanosome then they need to deal with uptake of natural ligands rather than artificial surrogates such as dextran. Such tracers were used historically, but in the last decade a series of receptors and ligands for fluid phase and particularly membrane mediated endocytosis have been discovered. With the investment of a little time these important ligand / receptors such as haptoglobin, transferrin, etc would be much more relevant.
Dextran is still state-of-the-art as it is an inert fluid phase marker. We are not aware - and have asked widely - of any readily-available alternative to dextran as a fluid phase marker, especially seeing as we have demonstrated in this study that BSA does not behave as a fluid phase marker in the experimental conditions used. The reviewer is also being disingenuous in suggesting that there is a panel of validated physiological reporters for trypanosomes that are readily available commercially - this is not the case. Transferrin is probably the only example, but the transferrin receptor is confined to the flagellar pocket and therefore not relevant to the question of how surface-bound material enters the flagellar pocket in the first place. As suggested by Reviewer 3 and endorsed by Reviewer 2, we have looked at the uptake of anti- VSG antibodies (which are a physiological cargo) in additional experiments and obtained evidence that the same effects are seen (Figure 9).

Reviewer #1 (Significance (Required)):

Unfortunately, I did not find tis to be very significant. It covers old ground in terms of the phenotype described. Many groups have shown the differences between procyclic and bloodstream phenotypes in this enlarged pocket phenomenon. The work is rather incremental from these and other author's work on these hook proteins.
There are alternative explanations for understanding the effect of flagella pocket structure and uptake of ligands into the pocket and trypanosome cell. These would need to be studied before one could see a functional, mechanistic link established.
Other parts of this are of nicely done but do not move on our understanding (eg targeting/phosphorylation) from what has been done previously.
As noted repeatedly, it appears that Reviewer 1's priority is disparaging the value of the work here and downplaying its significance rather than providing constructive feedback. The reviewer repeatedly makes unrealistic demands (a mechanistic model, use of non-standard reagents), misunderstands the aim of experiments (use of high-pressure freezing), makes vague allusions to

© 2023. Published by The Company of Biologists under the terms of the Creative Commons Attribution License (https://creativecommons.org/licenses/by/4.0/).

other work in the literature but without citing anything specific to support their case, and makes strong and assertive statements that are factually incorrect (design of RNAi experiments, use of dextran). We find this approach unhelpful, uncivil, and unprofessional. It is desperately disappointing that we should have to spend the majority of our response rebutting Reviewer 1's comments rather than implementing constructive criticisms that would strengthen the manuscript.

Referees cross-commenting

this session includes comments from Reviewer 1 and Reviewer 2.

Reviewer 2

Dear Reviewers 1 and 3:
I agree with many of the points with Reviewer 1 and our divergence is partly a matter of degree. While it is true that this manuscript is incremental in its contribution to our understanding of TbSmee1, it nonetheless adds to our understanding of the role of this protein in the bloodstream life stage and because of that I find value in the work. The fact that it mirrors what was seem in other protein knockdown studies (e.g. TbMORN) doesn't negate its contribution for me.
Reviewer 1 makes an important point, however, when stating that this work does not add a mechanistic or functional conclusion as to how TbSmee1 operates and for me that is the biggest shortcoming of the work. Offering mechanistic insight is a high bar and while it would make for a much more exciting story it does not discount the value of the work as presented. What I do appreciate is the speculation about this observation that endocytosis is required for entrance of surface bound material into the pocket and although they are unable to show that this is not a side affect of other processes being disrupted it is and intriguing point. These observation have the potential of stimulating further investigations into crosstalk between the entrance to the pocket and endocytosis. I also agree that the use of ligands for known receptors like transferrin would be far more informative. While I assumed the transferrin receptor was in the pocket itself it would be interesting to see if the ESAG6/7 is also located outside the pocket and transiently binds cargo before being brought inside for endocytosis.
I think that Reviewer 3 brings up a great point with the focus on VSG's. I think that examining VSG turnover in these mutants can add value to the analysis and inform our view of how affecting the hook complex alters VSG endocytosis.
We appreciate Reviewer 2 taking the time to defend the value of the work, and we concur with Reviewer 2's assessment. Reviewer 2 is also correct that the transferrin receptor appears to be primarily or wholly confined to the flagellar pocket interior, making this likely less informative in this context. Concerning the uptake of anti-VSG antibodies highlighted by Reviewer 3 and endorsed by Reviewer 2, we have carried out these experiments and obtained similar results to those published in the first version of the preprint (Figure 9).

Reviewer 1

Some fair comment and agreement. This is being sent to general cell biology journals. when one looks at this area in the round it is it is nearly 50 years (1975) since Langreth and Balber published their seminal work on protein uptake and digestion in bloodstream and culture forms of T. brucei. There has been 50 years intense study and the genome has been around for nearly 20 years as well. So, put simply - for both a general science audience and the wider parasite community - if this is a paper about one protein, TbSmee1,then it has surely has to say something functional about that protein. If it is a paper about uptake in trypanosomes (where mutants are one means of interrogation) then it surely has to say something about mechanisms of uptake of physiological relevant ligands. The days of dextran etc are past.
Hence, my comment that this does neither and so is very incremental to what is known already. It is 2022 not 1975. Langreth and Baber published their seminal work in J Protozoology for very good reasons no doubt.
It is striking that Reviewer 1 here extends their aggressive and uncivil approach to attack Reviewer 2's assessment, again substituting forceful wording for informed argument. Reviewer 1 again inexplicably and mistakenly criticises the use of dextran when no state-of-the-art alternative exists. They then go on to needlessly disparage the work done by Langreth & Balber when this work was produced in a totally different publishing landscape. They also appear to fundamentally

© 2023. Published by The Company of Biologists under the terms of the Creative Commons Attribution License (https://creativecommons.org/licenses/by/4.0/).

misunderstand the Review Commons concept, which is to provide journal- independent preprint peer review; it is also worth noting that there are specialist journals such as PLoS Pathogens in the RevComm affiliates as well as general cell biology journals. Given that the mechanism of variant surface glycoprotein (VSG) switching has not yet been fully articulated despite the efforts of multiple labs and many projects over a decades-long time period, it seems extremely unreasonable to be making such demands of this paper.

Reviewer 2
Thank you for replying and I agree with the spirit of your critique. My only comment, which could result from my own naivete, is to say that despite the incredible work that has been done in dissecting endocytosis in T. brucei over these past 50 years, it appears that we still do not understand how many fundamental of aspects of this activity works in this parasite. Even basic questions regarding how cargo, e.g. transferrin, binding to surface receptors is sensed by the parasite remains unknown and the identity of the specific signaling components which transmit this information internally to initiate endocytosis have not been characterized. In many ways it seems that we don't even understand how the parasite partitions the end/exocytic pathways in the pocket and maintains membrane homeostasis. While we know that some kinases and traditional signaling components must be involved, a high resolution understanding of this process in T. brucei seems lacking. I only say all this to suggest that the field maybe isn't yet that advanced to reject work of this type as so many mechanistic unknowns still remain to be uncovered and maybe incremental advances and phenomenology still can add value to the field. However, I respect your opinion on the matter and my perspective could be due to a lack of a full appreciation of the literature on the subject.
We completely agree with Reviewer 2's assessment here, which neatly summarises our rationale for the present work. Reviewer 2 is, if anything, being overly accommodating by suggesting that their perspective may be due to a lack of a full appreciation of the literature - on the contrary, Reviewer 2 appears to have a very sound grasp of the topic.

Reviewer #2 (Evidence, reproducibility and clarity (Required)):

Summary:
In this manuscript the authors have advanced our understanding of the hook complex component TbSmee1 through a detailed analysis of this protein's role in the endocytosis of surface bound proteins via the flagellar pocket in bloodstream form Trypanosoma brucei. The TbSmee1 protein, previously identified using proximity labeling using TbMORN1 and TbPLK, and characterized in procyclic T. brucei, was confirmed to target to both the shank portion of the hook complex as well as the growing end of the new FAZ in replicating cells. The protein was also shown to likely be phosphorylated as had been suggested previously due to its association with the kinase TbPLK. A domain deletion analysis demonstrated that domains 2 and 3 are important for TbSmee1's proper localization to the hook complex. Loss of TbSmee1 using RNAi based knockdown resulted in a quick cessation of growth in the bloodstream form within 24 hours in contrast to what was seen previously in procyclic cells which had only a decreased growth rate. Loss of TbSmee1 also resulted in an enlargement of the flagellar pocket and in many ways mirrored the phenotype observed with knockdown of TbMORN1. Although prior work on TbSmee1 in procyclic T. brucei demonstrated that loss of this protein altered the morphology of TbMORN1, no such change was seen in bloodstream form cells and only an alteration in the morphology of TbLRRP1 was observed. In characterizing the effect of TbSmee1 depletion on endocytosis the authors showed that the fluid phase marker Dextran could enter into the flagellar pocket of TbSmee1 depleted parasites while the surface bound ConA and BSA remained outside of the flagellar pocket suggesting that TbSmee1 may play a role in allowing larger protein components into the pocket regions. Similar observations were also previously seen with TbMORN1 depletion. Importantly, a knockdown of clathrin recapitulated the TbSmee1 knockdown phenotype suggesting that endocytosis itself was required to allow material bound at the surface to enter into the flagellar pocket. In addition to adding to our understanding of hook complex components, this work raises some interesting questions regarding the role of the hook complex in facilitating endocytosis in this important human pathogen.
Thank you for the positive assessment.

Major Critiques:
This is a superbly written manuscript with robust high-quality data that strongly support the major

© 2023. Published by The Company of Biologists under the terms of the Creative Commons Attribution License (https://creativecommons.org/licenses/by/4.0/).

conclusions made by the authors. The flow the article is logical and easy to follow making it accessible to a wide array of readers.
We are glad that the Reviewer appreciated the effort that went into writing the paper.

Although I appreciate the brevity of the introduction and how the article gets straight to the point, additional background information on the components and function of the flagellar pocket collar protein could help contextualize the goals of the project. The way in which the flagellar collar structures are introduced to the reader is quite abrupt (beginning on line 75) and simply states the names of TbBILBO1, the centrin arm and hook complex as simple facts without much discussion about the background of these components/regions. A graphical representation of the centrin arm or hook complexes relative to other components like the pocket itself, FAZ or axoneme could make following the story much easier. An expansion of this background could also go a long way to convince readers of the importance of this region in the basic biology and virulence of T. brucei.
Implemented. We have added more background details on the hook complex, flagellar pocket collar, and centrin arm and added a new schematic image to Figure 1 showing these structures as well as the FAZ (Figure 1A).

On lines 84-86 the authors cite the way in which 'small' vs 'large' macromolecules enter into the pocket without defining what exactly is meant by these terms as they are relative in nature. Setting some boundaries of size could provide some context to the reader.
Implemented. We have provided more detail on the approximate sizes in nm (lines 110-113 of tracked-changes manuscript).

In the domain localization analysis beginning in Figure 4 there is a missed opportunity to also assess which portions of the TbSmee1 protein are important for overall function as well. By either an examination of dominant negative phenotypes resulting from overexpression of the truncated mutant or the expression of the truncated forms designed to be RNAi resistant in the TbSmee1 knockdown cell line, one could also assess which portions of this protein are essential for endocytic function in addition to targeting. Is there a reason this was not performed?
This is a good point; we did actually investigate overexpression of the TbSmee1(161-766) construct which can target correctly but is missing the first folded domain, but did not observe any phenotypic effects. We have added this point to the results (lines 301-302 of tracked- changes version). We agree that it would be interesting to express the truncations in a TbSmee1 RNAi background in order to simultaneously assay for targeting and function, but this was (unfortunately, perhaps) not part of the original experimental design. To do so now would require generating a completely new panel of truncation constructs with recoded DNA (in order to make them RNAi-resistant) and then generating a new panel of cell lines. While this would be informative, we feel that it would be impractical at present.

In the analysis of viability changes due to TbSmee1 depletion (lines 237) the authors state that at "72 h post-induction showed widespread lysis, ..." This phenotype seems inconsistent with other related endocytic defect mutants. There is no further mention of this lysis phenomenon here or in the discussion and considering how unique this seems it deserves either additional data to demonstrate or further discussion as to the basis of the phenotype. It seems, at least from this study of TbStarkey1 and prior studies which result in the enlarged flagellar pocket phenotype, that having an enlarged pocket is not the cause of lysis and doesn't even naturally lead to a growth defect.
Widespread lysis is the usual outcome of bloodstream form cells with strong endocytic defects - we have observed this directly for the clathrin, TbMORN1, and TbSmee1 RNAi cell lines, and it has been documented in a number of other publications (see for example Natesan et al., 2010, Manna et al., 2017). We have clarified this point in the text (see for examples lines 359-341, 474-478 of tracked-changes manuscript).

The authors do not comment on what is the source for the cessation in growth following TbSmee1 knockdown. Is it nutrient depravation like in other endocytic defect mutants?
Implemented (see for example lines 359-361, 605-610 of the tracked-changes manuscript). The source of the growth defect is likely to be due to impaired cell division cycle progression due to the gross enlargement of the flagellar pocket and subsequent steric hindrance and imbalance of membrane homeostasis.

© 2023. Published by The Company of Biologists under the terms of the Creative Commons Attribution License (https://creativecommons.org/licenses/by/4.0/).

In the end, one of the most interesting observations made by the authors is that loss of TbSmee1 inhibits endocytosis and this has the appearance of not allowing large molecule substrates like ConA and BSA to enter into the flagellar pocket. This appeared to have nothing to do with a gatekeeping type function of the hook complex/flagellar collar and instead, as shown through clathrin knockdown, was related to the ability of the parasite to endocytose.
There are a lot of potential interpretations of this phenomenon with one being a simple perturbation of the normal membrane trafficking to and from the flagellar pocket being involved. An analysis of knockdown of exocytic components might reveal whether or not this inability to enter into the pocket is also seen when exocyst proteins are also depleted. It may be impossible to tease apart these two interrelated activities but it might eliminate one side of the equation if these proteins can still enter the flagellar pocket when exocytosis if perturbed although this reviewer understands that that dimension of T. brucei membrane trafficking is poorly understood relative to endocytosis.
This is an interesting point, and the reviewer is also correct in highlighting that exocytosis is far less characterised than endocytosis in Trypanosoma brucei. The exocyst has been characterised in bloodstream form T. brucei (Boehm et al., 2017) and shown to also have a role in endocytosis, so teasing out the relative contributions of these pathways would undoubtedly be challenging. We would prefer not to go in this direction in this present study, but it is an obvious avenue for future work.

An intriguing possibility that the authors allude to and which if answered would make this manuscript have a far broader appeal is to determine if loss of TbSmee1 alters the lipid kinase distribution and if this is the source of the negative impact on endocytosis. One important dimension of endocytosis in T. brucei which remains poorly understood is the role of signaling machinery in triggering endocytic events. It is possible that the hook complex serves as the gatekeeping or signaling platform that recruits signaling components (like lipid kinases) that identify and/or modify the membrane lipid phosphatidylinositols harboring cargo laden receptors thus marking them for endocytosis within the pocket. It still seems unclear when in the process of endocytosis is the decision made to pull things into the pocket but it seems that the assumption is that this occurs deep within the pocket. This data suggests that there is possibly another decision point prior to being allowed entrance into the pocket. It may be that this isn't a gatekeeping decision but rather a stop vs. go activity where once cargo laden membrane reaches the collar a choice is made to pull this material in or not there and not after material is already in the pocket.
These are all really interesting ideas and would be fascinating topics for future work.

This obvious enigma based on the observation that loss of hook complex components affect the spatially separated site of endocytosis support the idea that the actual endocytic signaling platforms are located at the hook complex and that this area may make the membrane modifications that mark membrane as being ready to be endocytosed via clathin coated vesicles at the bottom of the pocket. This would still allow for fluid phase small molecule entrance which does not require binding to surface proteins. The obvious problems of having both endo/exocytosis occurring in the same close proximity makes the dissection of this phenomenon difficult but it is worth potentially expounding on further in the discussion as this idea is very appealing and adds an important dimension to our understanding of endocytosis in this organism.
Implemented (lines 722-727 of the tracked-changes manuscript). We have added some more detail to these points in the Discussion. We agree with the reviewer that there are some profoundly interesting questions concerning membrane identify and membrane protein uptake here.

Minor Critiques:
The authors commit significant time to the analysis of the phosphorylation of TbSmee1, but there is little stated about the role of TbPLK in this activity or the potential connection of TbSmee1 phosphorylation to the cell cycle. Would a knockdown of TbPLK using RNAi potentially demonstrate an altered migration of TbSmee1 due to a lack of phosphorylation? An analysis of radiolabeled TbSmee1 using p32 in vivo would likely support this claim as well. Has mass spectrometry identified potential phosphorylation sites to examine? Additionally, the loss of TbSmee1 has been shown to disrupt localization of TbPLK in procyclic cells and so why this was not also assessed in bloodstream form cells subjected to RNAi was not clear.
Partly implemented. We have added some discussion of the possible role of TbSmee1 phosphorylation in the cell cycle to the Discussion (lines 562-565 of tracked-changes manuscript), and emphasised the identification of phosphorylation sites in previous phosphoproteomics work

© 2023. Published by The Company of Biologists under the terms of the Creative Commons Attribution License (https://creativecommons.org/licenses/by/4.0/).

(citations of Nett et al., 2009, Urbaniak et al., 2013). Given that the strongest and earliest effect of TbSmee1 depletion was on endocytosis and cargo uptake, we chose to focus on this angle rather than exploring its contribution to the biogenesis of cytoskeleton-associated structures and its interaction with TbPLK. For that reason we would prefer not to carry out the experiments looking at the effects of TbSmee1 depletion on TbPLK or vice versa.

In the results section (lines 104-108) a model of the protein structure as predicted for example by AlphaFold might be informative and complement the domain analysis work depending on the quality of the prediction.
Implemented. The AlphaFold prediction is consistent with the predictions made by the other structural analyses, and we have noted this in the text (lines 145-148 and 551 of the tracked-changes version).

There is an arrow in the Figure 1B Western blot but I can find no mention of what it is trying to highlight in the text.
Corrected.

For Figure 1D there is no loading control or control for the distribution of the soluble fraction to validate the separation of the two compartments.
Implemented. We have carried out additional experiments to show the partitioning of a cytoplasmic protein (the endoplasmic reticulum chaperone BiP) into the detergent-soluble fraction. These results are now displayed in the updated Figure 1.

The authors fail to comment on the lack of changes in hook complex components they see to that observed by Perry et. al. 2018. This difference merits some minor comment or speculation.
Implemented. We have added this commentary to the Discussion (lines 592-600 of the tracked-changes version).

Line 228: domain should be capitalized.
Implemented.

Line 230: FigS5C should have a space and period after Fig. and S5C.
Implemented.

Line 244: "on" should be inserted in the sentence "...TbSmee1 protein depletion ON either side of the onset..."
Implemented.

Line 400: the '...20/21 h post-induction...' is slightly confusing and may read better as 20-21 h.
Implemented.

Line 463: a space is needed between '...2009).The...'.
Implemented.

Reviewer #2 (Significance (Required)):

Significance:
This manuscript advances our current conception of endocytosis in T. brucei. Although this model kinetoplastid parasite has been extensively studied with respect to endocytosis there is still a great deal we do not yet understand regarding how this process is regulated at a mechanistic level. This work has begun to connect previously unappreciated aspects of endocytosis in T. brucei by highlighting a potentially novel connection between the flagellar collar/hook complex and the physically separated endocytic events within the flagellar pocket itself. It may be that what appears as regulated entrance into the pocket is in fact the source of signaling that triggers the endocytic events carried out by clathrin. This is an interesting notion that no doubt requires further investigation which lies outside of the scope of this report. While this work appeals primarily to those studying kinetoplastids parasites it has the potential to provide insight into basic protozoan biology as well. Due to my related interest in kinetoplastid endocytosis, I find this work to be of high quality, conceptually interesting and employs many of the cutting-edge techniques

© 2023. Published by The Company of Biologists under the terms of the Creative Commons Attribution License (https://creativecommons.org/licenses/by/4.0/).

currently available in the study of T. brucei.
We are very happy that the Reviewer formed a favourable impression of the work.

Reviewer #3 (Evidence, reproducibility and clarity (Required)):

This manuscript begins to dissect the function of the hook complex protein SMEE1 in the mammalian infective form of T. brucei. The hook complex is a cytoskeletal structure associated with the flagellar pocket, the only site of endo/exocytosis in these cells. The authors demonstrate that SMEE1 is required for endocytosis in these cells and that this can occur with minimal change to the molecular make-up of the hook complex. The authors show that endocytosis is important for the access of large molecules e.g. ConA into the flagellar pocket.

Major comments

The key conclusion of this study are convincing and the data is generally well presented and clear. The interpretation of the figures matches well with the data presented - there are a few minor issues though that I have highlighted below in minor comments. The authors use a range of molecular cell biology approaches to define the role of SMEE1 and these are appropriate and are well controlled.
Thank you.

My major comment focuses on the use of different tracers to study endocytosis but the elephant in the room is what is happening to VSG as this is the surface protein that needs to rapidly removed from the cell surface and cleaned. Given the importance of removal of antibodies bound to the VSG - have the authors looked at this in the SMEE1 depleted cells? Do VSG- antibody complexes accumulate in this region? This is an important experiment as this would give key physiologically relevant data to this study. All the material should be readily available for this as there are a number of VSG antibodies.
We agree with the Reviewer that the behaviour of these VSG-bound antibodies is a key test of the physiological relevance of the observations we have made using ConA and BSA, and have implemented this request - the results are in the new Figure 9. Although they sound simple, these assays turned out to be far from trivial and much more technically challenging than the other uptake assays, owing to the extremely fast kinetics (seconds) of anti-VSG uptake (Engstler et al., 2007) and the unexpectedly and incredibly high losses of bound antibodies during the assay. This might be due to shedding, as noted in the Discussion.

Minor comments

Perhaps I have been overthinking this but is surface-bound the right way to describe the cargo, as it clearly goes in both directions onto and off the surface and in fact the experiments in this manuscript are focussing on the removal of this material from the surface so is not surface- bound.
We have clarified that "surface-bound" refers to material that binds to the surface glycoprotein coat of the trypanosomes and which is subsequently internalised, not material that is bound for (i.e being directed to) the cell surface (lines 77-78 of tracked-changes version). We hope this addresses the Reviewer's point?

Have the authors investigated the structure of the protein using alphafold and if so how does that compare to the domain structure that was presented in this manuscript?
Implemented (lines 145-148, 551 of tracked-changes version). We have checked the AlphaFold prediction of the three-dimensional structure of TbSmee1 and noted it in the Results; the prediction is consistent with the earlier bioinformatic analyses.

The authors raised a number of antibodies to TbSMEE1 and TbSTARKEY1 but it was not clear in the figures which antibody was ultimately used for analysis by western and IF - could the authors clarify, as some looked to have a higher background than others. Line 150 states the same localisation was seen for all three antibodies and references S3C but I couldn't see that data presented.
Implemented - the 304 antisera was used for most subsequent experiments and we have noted this in the M&M (lines 793-798 of tracked-changes version). Figure S3C shows that the Ty1- TbSmee1 recapitulates the localisation of the antibodies against the endogenous protein - we have clarified

© 2023. Published by The Company of Biologists under the terms of the Creative Commons Attribution License (https://creativecommons.org/licenses/by/4.0/).

this point as well (lines 206-207 of tracked-changes version).

Line 169 - can the authors provide more detail about the global correlation methodology as I was unable to follow the details in the methods? Is this a pixel per pixel correlation over the image or on a selected region over the area of potential signal overlap? In figure 2E it appears that BILBO1 signal correlates more closely with the SMEE1 signal than MORN and LRRP1 and from the images that would not seem to be the case. Have I interpreted this figure incorrectly?
Implemented. The original analysis was a global correlation analysis that was determining whether the signals were correlated with each other regardless of spatial overlap, and we agree with the reviewer that these outputs were non-intuitive to interpret. In the revision, we have carried out a new analysis (and updated the accompanying text and M&M section), measuring the degree of spatial correlation between each pair of signals on a pixel-by-pixel basis over the area of each cell, with a total of 30 cells analysed in each pairing. We believe that this addresses the reviewer's point. See lines 223-243, 963-974 of the tracked-changes version).

The authors have generated a number of different clones and performed experiments on these clones generally more than twice, which is clearly explained in the figure legends but in places the data is then put together and it is difficult to know which experiments/clones it comes from - for example 7C/7F what do those percentages represent? Is this the sum of all experiments? A representative experiment? How many cells per experiment were analysed?
Implemented. We have double-checked all the figure legends and clarified this point where necessary. Quantifications were always made by compiling data from multiple independent experiments using multiple separate clones - see in particular lines 1323-1324, 1363-1365, 1380-1382 of the tracked-changes version.

Line 200 - From the image it is not convincing that SMEE1 is slightly behind DOT1 - I agree it looks enveloped but would appear level with the distal end of the DOT1 signal.
Implemented. We have adopted the Reviewer's wording for this text (line 271 of tracked- changes version).

For the truncation experiments the authors should explain that these are performed with cells in which the endogenous SMEE1 will be expressed and this may influence the localisation of the truncations, especially as there is no information about whether SMEE1 forms complexes with itself or other proteins.
Implemented (lines 296-298 of tracked-changes version).

Figure 4D - should be 1 not T-
We have relabelled this as "TbSmee1". The values in this column are the immunoblot signal intensities obtained for the endogenous TbSmee1 protein in the -Tet condition. We have also clarified this in the figure legend.

Line 223 - given the low expression of constructs 2 and 9 I'm not sure it is possible to infer anything from the lack of localisation of these constructs as they appear unstable and would be unlikely to localise to a specific location.
We have added this caveat to the text (lines 558-562 of tracked-changes version).

Figure S7 - The images presented were not convincing that there was a reduction in the localisation of LRRP1 to the hook complex on depletion of either SMEE1 or MORN1. The difference looks particularly minor if present at all.
Agreed, there was some debate in the group about these results. We have changed to text to fit the Reviewer's interpretation (lines 347-348 of the tracked-changes version).

Line 264 - "implied that the lethal phenotype might be due to a loss of function" - this seems an odd thing to say as it doesn't provide any insight as of course the phenotype is due to a loss of function.
We have clarified this point (lines 350-353 of the tracked-changes version). We would however disagree with the reviewer that RNAi phenotypes are exclusively due to a loss of individual protein's function(s) - when proteins are present in multiprotein complexes (as is often the case with cytoskeleton-associated proteins), then destabilisation of the complex due to loss of the entire protein can cause the observed phenotype, rather than the loss of the function performed by the individual protein within the complex (this may be a semantic point, however). A very good

© 2023. Published by The Company of Biologists under the terms of the Creative Commons Attribution License (https://creativecommons.org/licenses/by/4.0/).

example of this is with the outer arm dynein complex component LC1 (Ralston et al., 2011) - RNAi against LC1 is lethal because the entire outer arm dynein complex is destabilised, whereas expression of non-functional mutants of LC1 produces viable cells with motility defects due to the specific loss of LC1 function.

Line 412 - can the authors clarify what they mean by geometric problems?
Implemented (lines 605-610 of tracked-changes version). We were referring to the fact that enlargement of the flagellar pocket will probably create difficulties for the progression of the cell division cycle.

Throughout the manuscript can you use log scale for the growth curves.
Implemented.

Line 756 - add citation
Whoops! Implemented (line 1058 of tracked-changes version).

Line 465/66 - the authors states that the ability of the fluid phase cargo being still able to enter the pocket is evidence that the channel lumen is still open; however, I would think that despite the close apposition of the cell membrane to the flagellar membrane in the flagellar pocket neck region this would be unlikely to impede fluid/soluble material from entering the pocket, as presumably VSG protein can move through this region. This does not alter the ultimate conclusion the authors are drawing but without microscopy evidence for the state of the channel lumen it is difficult to be sure of its status.
Fair point. We have modified this statement (line 701 in tracked-changes version).

Reviewer #3 (Significance (Required)):

The flagellar pocket is the key portal into and out of the trypanosome cell and as such has a vital role to play in host-parasite interactions. The flagellar pocket is supported by a number of cytoskeletal structures including the hook complex and the role of these structures in flagellar pocket function are poorly understood. The flagellar pocket is particularly important in the bloodstream form of the trypanosome parasite which infects the mammalian host as it is the route for the surface protein VSG to get onto and off the surface. The VSG is required for antigenic variation and the removal of VSG-antibody complexes helps 'clean' the surface of the parasite. SMEE1 is a component of the hook complex and the manuscript here dissects its role in the mammalian infective parasite and shows that it is vital for the endocytosis of material off the surface. Intriguingly, a block in endocytosis causes a blockage of material outside of the pocket, suggesting a multi-step process in the regulation of uptake of material from the parasite's surface. This manuscript will be of specific interest to those researchers investigating the long-term persistence of these parasites in the mammalian host. There are potentially some insights into the control of membrane domains for endocytosis that are of interest to more general cell biologists as well.
We are very grateful to the reviewer for the supportive comments and the constructive evaluation. Many thanks!

Expert in molecular cell biology of trypanosomes and Leishmania.

First decision letter

MS ID#: JOCES/2023/261548
MS TITLE: Characterisation of TbSmee1 indicates that endocytosis is required for access of surface-bound cargo to the trypanosome flagellar pocket
AUTHORS: Daja Schichler, Antonia Konle, Eva-Maria Spath, Sina Riegler, Alexandra Klein, Anna Seleznev, Sisco Jung, Timothy Wuppermann, Noah Wetterich, Alyssa Rossi Borges, Elisabeth Meyer-Natus, Katharina Havlicek, Sonia Perez Cabrera, Korbinian Niedermueller, Sara Sajko, Maximilian

© 2023. Published by The Company of Biologists under the terms of the Creative Commons Attribution License (https://creativecommons.org/licenses/by/4.0/).

Dohn, Xenia Malzer, Emily Riemer, Tuguldur Tumurbaatar, Kristina Djinovic-Carugo, Gang Dong, Christian J Janzen, and Brooke Morriswood
ARTICLE TYPE: Research Article

Thank you for sending your manuscript to Journal of Cell Science through Review Commons.

I am happy to tell you that after your revision your review commons manuscript has been accepted for publication in Journal of Cell Science, pending standard ethics checks.

© 2023. Published by The Company of Biologists under the terms of the Creative Commons Attribution License (https://creativecommons.org/licenses/by/4.0/).
